# NF-κB-induced microRNA-31 promotes epidermal hyperplasia by repressing protein phosphatase 6 in psoriasis

**DOI:** 10.1038/ncomms8652

**Published:** 2015-07-03

**Authors:** Sha Yan, Zhenyao Xu, Fangzhou Lou, Lingyun Zhang, Fang Ke, Jing Bai, Zhaoyuan Liu, Jinlin Liu, Hong Wang, Huiyuan Zhu, Yang Sun, Wei Cai, Yuanyuan Gao, Bing Su, Qun Li, Xiao Yang, Jianxiu Yu, Yuping Lai, Xue-Zhong Yu, Yan Zheng, Nan Shen, Y. Eugene Chin, Honglin Wang

**Affiliations:** 1 Shanghai Institute of Immunology, Key Laboratory of Cell Differentiation and Apoptosis of Chinese Ministry of Education, Shanghai Jiao Tong University School of Medicine (SJTU-SM), Shanghai 200025, China; 2 Shanghai Institute of Hypertension, Ruijin Hospital, Shanghai Jiao Tong University School of Medicine (SJTU-SM), Shanghai 200025, China; 3 State Key Laboratory of Proteomics, Genetic Laboratory of Development and Diseases, Institute of Biotechnology, Beijing 100071, China; 4 Shanghai Key Laboratory for Tumor Microenvironment and Inflammation, Shanghai Jiao Tong University School of Medicine (SJTU-SM), Shanghai 200025, China; 5 Shanghai Key Laboratory of Regulatory Biology, School of Life Sciences, East China Normal University, Shanghai 200241, China; 6 Department of Microbiology & Immunology, Medical University of South Carolina, Charleston, South Carolina 29425, USA; 7 Department of Dermatology, The Second Affiliated Hospital of Xi'an Jiaotong University, Xi'an 710004, China; 8 Institute of Health Sciences, Shanghai Institutes for Biological Sciences, Chinese Academy of Sciences/Shanghai Jiao Tong University School of Medicine (SJTU-SM), Shanghai 200025, China

## Abstract

NF-κB is constitutively activated in psoriatic epidermis. However, how activated NF-κB promotes keratinocyte hyperproliferation in psoriasis is largely unknown. Here we report that the NF-κB activation triggered by inflammatory cytokines induces the transcription of microRNA (miRNA) miR-31, one of the most dynamic miRNAs identified in the skin of psoriatic patients and mouse models. The genetic deficiency of miR-31 in keratinocytes inhibits their hyperproliferation, decreases acanthosis and reduces the disease severity in psoriasis mouse models. Furthermore, protein phosphatase 6 (ppp6c), a negative regulator that restricts the G1 to S phase progression, is diminished in human psoriatic epidermis and is directly targeted by miR-31. The inhibition of ppp6c is functionally important for miR-31-mediated biological effects. Moreover, NF-κB activation inhibits ppp6c expression directly through the induction of miR-31, and enhances keratinocyte proliferation. Thus, our data identify NF-κB-induced miR-31 and its target, ppp6c, as critical factors for the hyperproliferation of epidermis in psoriasis.

## Abstract (web summary)

Psoriasis is accompanied by NF-κB activation and hyperplasia. Here the authors show that NF-κB transcriptionally activates miR-31, which downregulates a negative cell cycle regulator protein phosphatase 6, and that this is critical for NF-κB to drive keratinocyte hyperproliferation.

## Graphical abstract

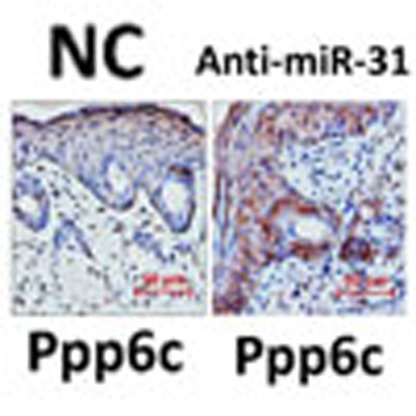

Psoriasis is a complex, chronic inflammatory disease affecting the skin among 2–3% of the general population^1^. Psoriasis is manifested as white silvery scales covered with erythematous plaques, and it is a lifelong disorder that severely reduces the quality of life of those affected^2^. Psoriatic lesions are characterized by epidermal hyperplasia with loss of the superficial granular layer, thickening of the cornified envelope, aberrant differentiation of keratinocytes and a dramatic infiltration of the major inflammatory immune cells into the dermis or epidermis^3^. It is now widely accepted that a dysregulated crosstalk between epidermal keratinocytes and immune cells leads to epidermal hyperplasia in psoriasis, and NF-κB may act as a link in this crosstalk^3^,^4^.

NF-κB is sequestered by its inhibitor IκB in the cytoplasm of resting cells as a transcriptionally inactive form^5^. Once dissociated from IκB, p65 undergoes phosphorylation, enters the nucleus and initiates transcriptional activity^6^. In sharp contrast to the absence of phosphorylated p65 in the epidermis of normal skin, the epidermis of psoriatic plaques exhibits a high level of phosphorylated p65, closely correlating with the grade of epidermal hyperplasia^7^,^8^. Moreover, the tumour necrosis factor-α (TNF-α)-targeting agent etanercept markedly inhibits p65 phosphorylation in the epidermal compartment, which is accompanied with an attenuation of epidermal thickness, restoration of keratinocyte differentiation molecular indicators and favourable clinical outcomes of psoriasis patients^7^. These studies strongly suggest a critical role of epidermal NF-κB activation in the pathophysiology of the disease.

Several factors including A20 of the NF-κB signalling pathway are genetically linked to psoriasis as revealed by genome-wide association studies. Located in the cytoplasm, A20 is a zinc finger protein encoded by *TNFAIP3* that regulates the NF-κB pathway via triggering IKKγ destruction^9^. *TNIP1*, *NFKBIA* and *TRAF3IP2* encoding the NF-κB regulatory proteins ABIN, IκBα and ACT1, respectively, were reported to be associated with psoriasis^10^,^11^,^12^. Recently, a multi-center, case-control study associated psoriasis and psoriatic arthritis with several rare missense mutations in *CARD14,* which is localized within keratinocytes and exerts regulatory effects on NF-κB^13^. Despite the importance of the activated NF-κB pathway in epidermal hyperplasia of psoriasis, the critical intrinsic factor(s) that triggers basal keratinocyte hyperproliferation in the downstream of NF-κB signalling is not well-defined.

MicroRNAs (miRNAs) are single-stranded, noncoding short RNA molecules regulating gene expression by binding target(s) of complementary messenger RNAs (mRNAs) and inhibiting their expression via interruption of protein translation and mRNA degradation^14^. Previous studies reported a distinct miRNA expression profile in psoriatic skin compared with healthy skin, and these deregulated miRNAs have been suggested to regulate keratinocyte proliferation and/or differentiation or suppress T-cell apoptosis in psoriasis^15^,^16^,^17^,^18^,^19^,^20^. More recently, an interesting study showed that overexpressed miR-31 is present in psoriatic keratinocytes and contributes to psoriatic inflammation by modulating inflammatory mediator production and leucocyte infiltration to skin^21^. Nevertheless, the physiological significance and the *in vivo* function of endogenous miR-31 in basal keratinocytes in the epidermal hyperplasia of psoriasis remain poorly understood.

Here we show that the inflammatory cytokines that activate NF-κB signalling in keratinocytes induce the NF-κB-dependent transcription of miR-31 in the epidermis of lesional skin derived from not only psoriatic mouse models but also patients with psoriasis. We demonstrate a previously unrecognized role of miR-31 in regulating the keratinocyte cell cycle by generating a knockout mouse model with a conditional deletion of miR-31 in epidermal basal keratinocytes. We have revealed that the miR-31 deletion in basal keratinocytes inhibits acanthosis and reduces the disease severity in two mouse models of psoriasis. Moreover, we show that protein phosphatase 6 (ppp6c), an inhibitor of the G1–S phase transition in the cell cycle, is diminished in epidermis derived from human psoriatic skin and is directly targeted by miR-31. Ppp6c inhibition is functionally essential for the biological effects mediated by miR-31 in epidermal hyperplasia. Taken together, our results support the existence of a post-transcriptional mechanism through which NF-κB signalling modulates the hyperproliferation of basal keratinocytes and identify NF-κB-induced miR-31 and its target ppp6c as important factors in the development of chronic inflammatory skin disorder.

## Results

### miR-31 expression is elevated in psoriatic skin

**Figure 1:**
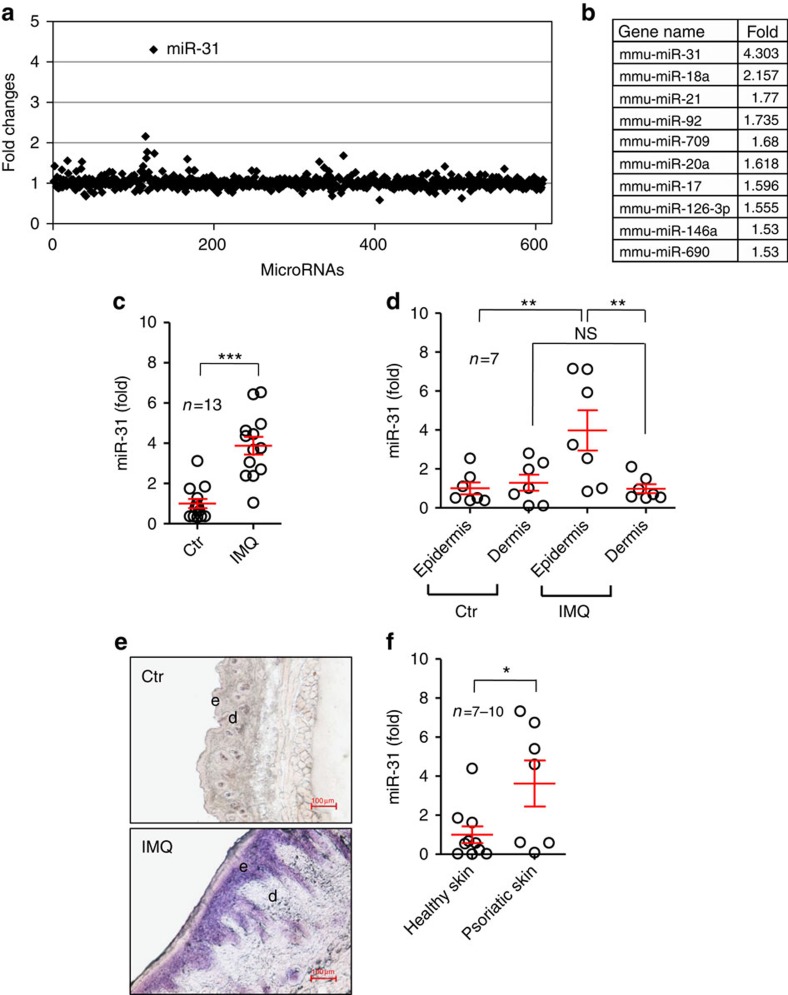
Expression of miR-31 in lesional skin of psoriasis mouse models and patients. (**a**) Screening for miRNAs that are differentially expressed in lesions derived from the CD18^hypo^ PL/J mouse model of psoriasis. Fold changes in the expression levels of 610 miRNAs between affected CD18^hypo^ PL/J and control CD18 WT PL/J mice are shown. (**b**) miRNAs upregulated in affected ears of CD18^hypo^ PL/J mouse model of psoriasis (>1.5-fold). (**c**) Expression of miR-31 in healthy skin samples from untreated mice and in lesional skin samples from IMQ-induced mouse model. (**d**) Expression of miR-31 in the epidermis and dermis of healthy skin from untreated controls or lesional skin from IMQ-treated mice. (**e**) *In situ* hybridization was performed on mouse skin treated with vehicle (upper panel, *n*=6) or IMQ (lower panel, *n*=5) using miR-31-specific LNA probe. Purple colour indicates miR-31 expression. e, epidermis; d, dermis; scale bar, 100 μm. (**f**) Expression of miR-31 in healthy human skin and psoriatic human skin samples. Results (**c**,**d**,**f**) are presented as the ratio of miRNA to the small nuclear RNA U6, relative to that in untreated mice (**c**,**d**) or relative to that in healthy skin samples (**f**). *P<0.05, **P<0.01, ***P<0.001, two-tailed Student's *t*-test. Data (**c**–**f**) are representative of two or three independent experiments with 5–13 samples per group in each (mean and s.e.m.). Ctr, untreated mice; IMQ, IMQ-treated mice.

To identify miRNAs that are preferentially expressed in inflamed keratinocytes, we carried out comparative miRNA screening of normal ear skin from control mice and of lesional ear skin from CD18 hypomorphic (CD18^hypo^) mice, which spontaneously develop a T-cell-mediated psoriasiform skin disease^22^. Of the 610 miRNAs analysed, we observed that miR-31 was the miRNA that was most highly expressed in affected skin (4.3-fold increase) (Fig. 1a,b). The increased expression of miR-31 in diseased skin promoted us to assess its *in vivo* functionality in an imiquimod (IMQ)-induced psoriasis mouse model that closely resembles the human disease phenotype^23^. To perform this assessment, we painted Aldara, a cream containing IMQ on the back of shaved mice for 7 consecutive days. In agreement with other study^23^, both C57BL/6J and BALB/c mice treated with IMQ developed sharply demarcated erythematous lesions covered with white silvery squama (Supplementary Fig. 1a,b). Hyperplasia of the epidermis with regular elongation of rete ridges, hyperkeratosis, increased proliferative basal layer epidermal keratinocytes, dilated capillaries, microabscesses and massive dermal cellular infiltrates were histologically evident in the IMQ-treated mouse skin (Supplementary Fig. 1c). Notably, by quantitative real-time PCR (qPCR), we determined that miR-31 was significantly increased in the lesional skin from IMQ-treated mice compared with the healthy skin (1±0.2276 versus 3.877±0.4426, *n*=13, *P*<0.0001, two-tailed Student's *t*-test) (Fig. 1c), and the upregulated miR-31 was mainly confined to epidermis (Fig. 1d). To confirm this observation, we performed *in situ* hybridization on skin cryosections from the IMQ-treated mice using miR-31-specific locked nucleic acid-modified (LNA) probes. We found that miR-31 expression was restricted to the basal and suprabasal cell layers of the epidermis in the lesional skin of the IMQ-treated mice (Fig. 1e). Consistent with a published report^21^, we showed that miR-31 expression was significantly upregulated in lesional skin from patients with psoriasis (Fig. 1f). Taken together, these data demonstrate that the expression of miR-31 is abundantly increased in the affected skin of psoriasis patients and mouse model, and support the notion that overexpressed miR-31 in hyperproliferating keratinocytes may be functionally involved in the pathogenesis of psoriasis.

### NF-κB activation induces miR-31 expression

**Figure 2:**
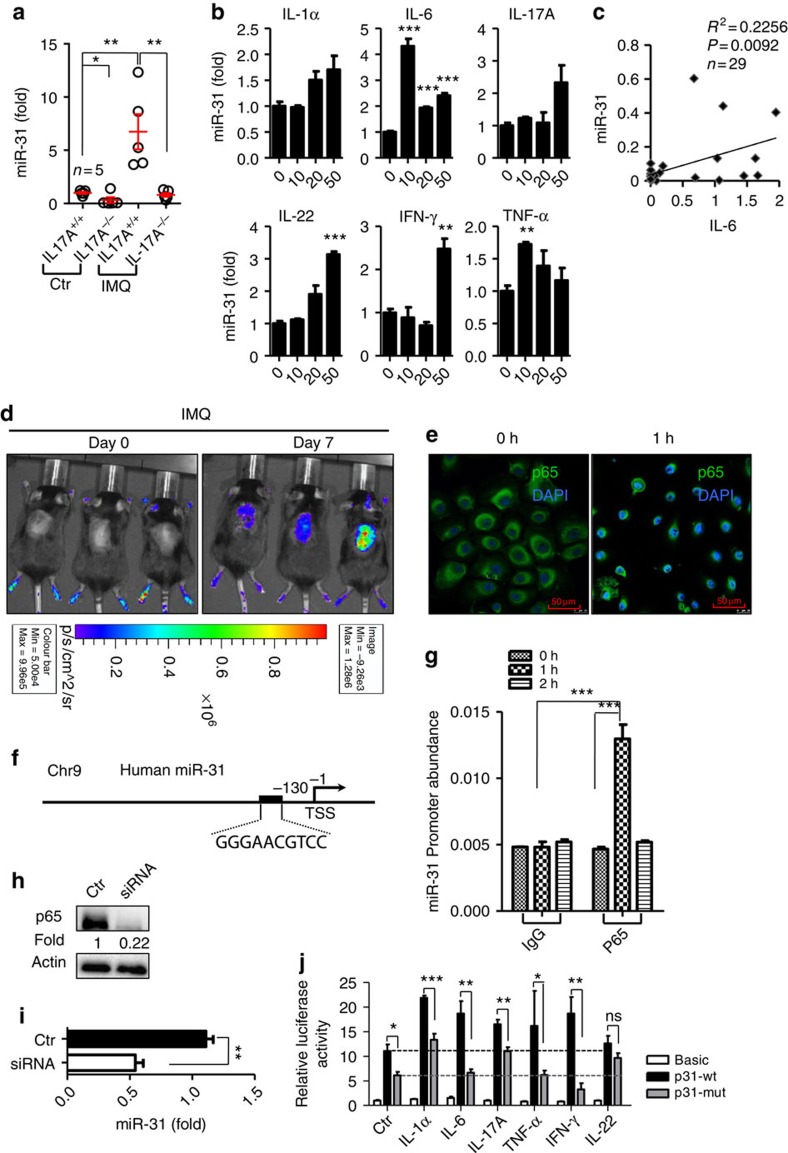
Requirement of NF-κB signaling for the induction of miR-31 in epidermal keratinocytes. (**a**) miR-31 expression in the epidermis of skin samples derived from either IL-17A^+/+^ or IL-17A^−/−^ mice without treatment or after treatment with IMQ. (**b**) miR-31 expression in NHEK stimulated with 0 ng ml^−1^, 10 ng ml^−1^, 20 ng ml^−1^ and 50 ng ml^−1^ of IL-1α, IL-6, IL-17, IL-22, IFN-γ and TNF-α for 24 h. (**c**) Correlation between IL-6 and miR-31 expression in 29 psoriatic skin samples. (**d**) The NF-κB-driven luciferase activity was monitored at day 0 and day 7 after the application of IMQ in NF-κB luciferase reporter mice. (**e**) NHEK were stimulated with IL-6 for 0 and 1 h. The p65 nuclear translocation was imaged at 0 and 1 h. Scale bar, 50 μm. (**f**) The schematic diagram showed one potential binding sites of p65 in the putative promoter element of human miR-31. (**g**) Phosphorylated p65 was immunoprecipitated from NHEK stimulated with IL-6. Immunoprecipitates were assayed for the expression levels of miR-31 promoter. (**h**) NHEK were transfected with scramble siRNA (Ctr) or p65 siRNA (siRNA). Cell lysates were immunoblotted with anti-p65 or anti-actin. Values were expressed as fold changes relative to controls and normalized to β-actin. (**i**) miR-31 expression in NHEK transfected with scramble siRNA (Ctr) or p65 siRNA (siRNA p65), and stimulated with IL-6 for 24 h. (**j**) Luciferase activity in lysates of HaCaT keratinocytes transfected with luciferase reporter plasmids of pGL3-basic empty vector (basic), miR-31 promoter (p31-wt) or miR-31 promoter with mutation on the predicted NF-κB binding site (p31-mut), unstimulated or stimulated with indicated cytokines. Results are presented as the ratio of firefly luciferase to renilla luciferase activity, relative to that of unstimulated HaCaT keratinocytes transfected with pGL3-basic empty vector. Black or grey dotted line indicates the mean of relative luciferase activity in unstimulated HaCaT keratinocytes. Results (**a**,**b**,**i**) are presented as the ratio of miRNA to the small nuclear RNA U6, relative to that in untreated IL-17A^+/+^ mice (**a**) or relative to that in non-stimulated keratinocytes (**b**) or relative to that in siRNA control-treated keratinocytes (**i**). *P<0.05, **P<0.01, ***P<0.001, NS, not significant, two-tailed Student's *t*-test. Data are representative of at least two independent experiments with four to five samples per group in each (mean and s.e.m.).

The expression of Th17 cytokines such as interleukin-17 (IL-17) and IL-22 is elevated in psoriatic skin^24^, and anti-IL-17 agents are clinically effective in the treatment of human psoriasis^25^,^26^. We sought to investigate whether the upregulation of Th17 cytokines coincides with the induction of miR-31 in skin inflammation. We compared the miR-31 induction in lesional skin derived from IL-17A-deficient (IL-17A^−/−^) and wild-type (IL-17A^+/+^) mice treated with IMQ. Clearly, miR-31 expression was significantly decreased in the psoriatic lesions in the IL-17A^−/−^ mice compared with the IL-17A^+/+^ mice after the IMQ application (Fig. 2a). To examine the possible involvement of key inflammatory cytokines in the induction of miR-31, we stimulated primary normal human epidermal keratinocytes (NHEK) with IL-1α, IL-6, IL-17A, IL-22, interferon-γ (IFN-γ) and TNF-α. Almost all of the tested inflammatory cytokines were able to stimulate miR-31 expression to different extents; however, IL-6 appeared to be an inducer of miR-31 at low concentrations in NHEK (Fig. 2b). Thus, we chose IL-6 as a stimulus for miR-31 induction. Interestingly, we further demonstrated that IL-17A deletion led to a pronounced decrease of IL-6 expression in the epidermis of the mouse lesional skin induced by IMQ (Supplementary Fig. 2a), suggesting that IL-17A is a proinflammatory cytokine that modulates IL-6 production in an IMQ-induced mouse model. Importantly, we observed that IL-6 expression was closely correlated with miR-31 expression in human psoriatic lesions (Fig. 2c and Supplementary Table 1). Although activation of the STAT3 transcription factor is a classical downstream event of IL-6 stimulation, we found that there was no significant difference of the miR-31 levels in the HaCaT keratinocytes stimulated with IL-6 in the absence and presence of a STAT3 inhibitor (Supplementary Fig. 2b), indicating that the miR-31 expression induced by IL-6 may not be mediated by STAT3 signalling. NF-κB activation plays an essential role in regulating miRNAs^27^. In the intestinal epithelia, IL-6 has also been reported to induce NF-κB activation^28^. We sought to investigate whether IL-6 could trigger the expression of miR-31 mediated by NF-κB signalling in keratinocytes. We applied IMQ to the back skin of NF-κB luciferase reporter mice thus enabling *in vivo* real-time imaging of NF-κB activity. Indeed, NF-κB-driven luciferase activity was markedly increased in the exposed areas applied with IMQ (Fig. 2d), suggesting that the NF-κB signalling pathway is activated in affected skin in the IMQ-induced mouse model. Flow cytometry analysis confirmed that the IMQ-induced NF-κB activation was in keratinocytes rather than CD45^+^ leucocytes (Supplementary Fig. 2c). Moreover, the nuclear translocation of the p65 NF-κB subunit was observed in NHEK stimulated with IL-6 for 1 h (Fig. 2e). A database analysis identified one potential p65-binding site in the promoter element at −130 upstream from the transcription start site of human miR-31 (Fig. 2f). To verify the binding of the p65 component of NF-κB to the putative binding site in the promoter element of miR-31, we performed chromatin immunoprecipitation (ChIP) assays. These assays showed that IL-6 stimulation resulted in the recruitment of p65 to the putative miR-31 promoter (Fig. 2g). Using a specific short interfering RNA (siRNA), we further silenced p65 in NHEK (Fig. 2h). In contrast to the controls, the knockdown of p65 led to a significant decrease in miR-31 expression in keratinocytes stimulated with IL-6 (Fig. 2i). To further confirm whether IL-1α, IL-6, IL-17A, TNF-α, IFN-γ and IL-22 are able to induce the expression of miR-31 through NF-κB signalling, we used luciferase reporter constructs driven by miR-31-specific promoter response in HaCaT keratinocytes. We found that IL-1α, IL-6, IL-17A, TNF-α, IFN-γ and IL-22 stimulation increased p65 binding to the putative binding site at −130 of the putative promoter of miR-31, and mutation of the binding site at −130 blocked the luciferase activity induced by these cytokines (Fig. 2j). Thus, we suggest that IL-1α, IL-6, IL-17A, TNF-α, IFN-γ or IL-22 directly or indirectly activates the NF-κB signalling pathway mediating miR-31 expression in keratinocytes.

### miR-31 conditional deletion reduces the disease severity

To further study the potential role of miR-31 in the aetiology and pathogenesis of psoriasis, we created miR-31 transgenic mice (miR-31^TG^) using a viral vector carrying enhanced green fluorescent protein (EGFP) and pri-miR-31 under the control of the cytomegalovirus (CMV) promoter (Supplementary Fig. 3a,b). Compared with the wild-type (WT) littermates, the miR-31^TG^ mice increased miR-31 by 4.0-fold (1±0.4895 versus 4.078±1.082, *n*=4–5, *p*=0.0498, two-tailed Student's *t*-test) (Supplementary Fig. 3c). The magnitude of this increase is similar to what we observed in psoriatic lesions.

**Figure 3:**
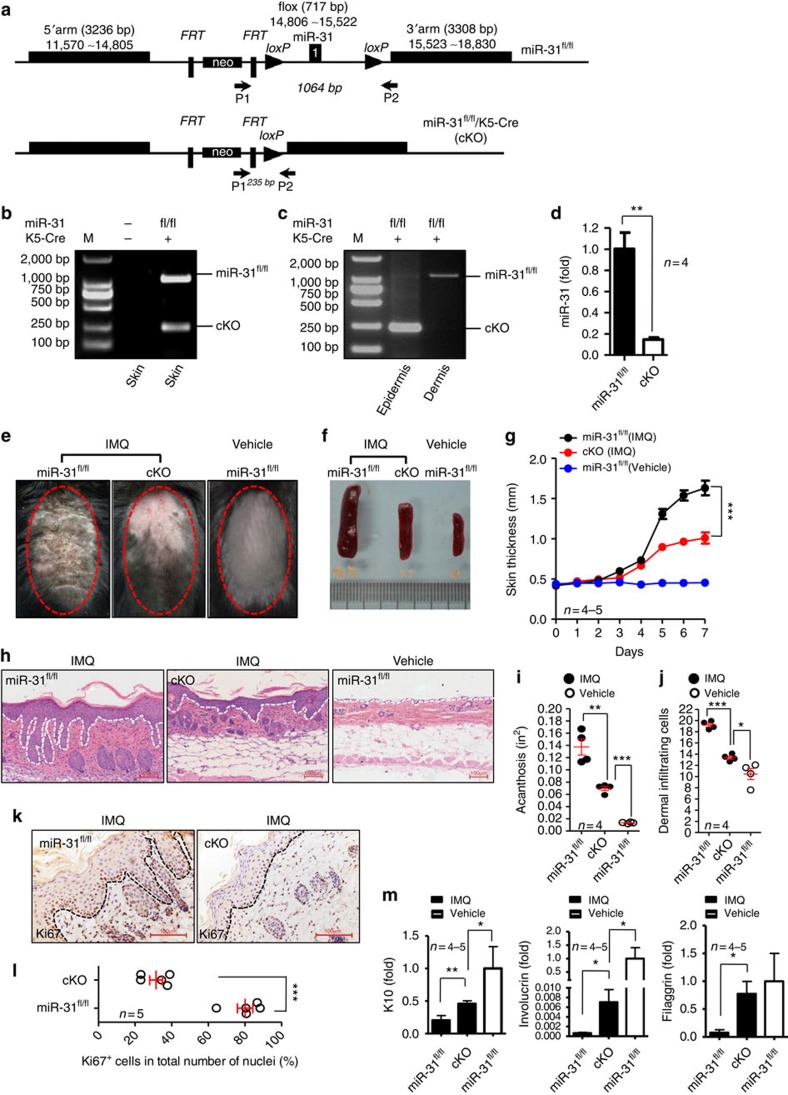
Decreased epidermal hyperplasia and dermal cellular infiltrates in miR-31^fl/fl^/K5-Cre mice treated with IMQ. (**a**) Schematic representation of primers for genotyping and targeting strategy. (**b**) miR-31 genotyping using P1/P2, 1,064 bp band for miR-31^fl/fl^ and 235 bp band for miR-31^fl/fl^/K5-Cre (cKO). DNA samples were prepared from total skin. (**c**) Cre-mediated tissue-specific deletion of miR-31 in epidermis. DNA samples were prepared from either epidermis or dermis. (**d**) miR-31 expression in epidermis derived from miR-31^fl/fl^ and cKO mice. (**e**) Phenotypic presentation of mouse back skin for miR-31^fl/fl^ or cKO mice treated with IMQ or vehicle for 7 days. (**f**) Splenomegaly and lymphadenopathy in miR-31^fl/fl^ or cKO mice treated with IMQ or vehicle for 7 days. Data are representative of more than five mice. (**g**) Skin thickness was measured on the days indicated. Symbols represent mean skin thickness±s.e.m. for five to six mice per group. (**h**) H&E staining of the back skin of miR-31^fl/fl^ or cKO mice treated with IMQ or vehicle. Dotted line indicates the border between the epidermis and the dermis. Scale bar, 100 μm. (**i**) Acanthosis of miR-31^fl/fl^ or cKO mice treated with IMQ or vehicle. (**j**) Dermal cellular infiltrates of miR-31^fl/fl^ or cKO mice treated with IMQ. (**k**) Immunostaining of Ki67 in lesional skin derived from miR-31^fl/fl^ (left panel) or cKO (right panel) mice treated with IMQ. Scale bar, 100 μm. (**l**) Quantitation of Ki67^+^ cells in epidermis. For all measurements, numbers of specifically stained Ki67^+^ cells in epidermis counted in three high-power fields for each section were used. (**m**) qPCR analysis of epidermal differentiation markers (Keratin 10, Loricrin and Filaggrin) in miR-31^fl/fl^ or cKO mice treated with IMQ or vehicle. Results (**d**,**m**) are presented as the ratio of miRNA to the small nuclear RNA U6 or of mRNA to the β-actin, relative to that in miR-31^fl/fl^ mice. *P<0.05, **P<0.01, ***P<0.001, two-tailed Student's *t*-test for **d**,**i**,**j**,**l**,**m**, one-way ANOVA for **g**. Data (**g**,**i**,**j**,**l**,**m**) are representative of two independent experiments with four to six mice per group in each (mean and s.e.m.).

We next investigated the role of miR-31 in the development of IMQ-induced psoriasiform skin disease, and found that the miR-31^TG^ mice displayed a more severe form of the disease than the control mice (Supplementary Fig. 4a–d). Moreover, we detected that NHEK exhibited an enhanced proliferation after overexpressing miR-31 (Supplementary Fig. 5). To further investigate the functional relevance of miR-31 with psoriasiform skin inflammation in a loss-of-function setting, we used homologous recombination for generating mice with a *miR-31* allele flanked by *loxP* sites (floxed, Fig. 3a). The germline-transmitted mice were further crossed with Keratin 5-Cre transgenic mice to achieve a conditional knockout mouse model with a deleted *miR-31* allele in the epidermal basal keratinocytes (Fig. 3b–d). The skin of the miR-31^fl/fl^/Keratin 5-Cre (cKO) mice remained healthy without any detectable inflammatory pathology for at least 32 weeks. We then applied IMQ to both the miR-31 control (miR-31^fl/fl^) and cKO mice, and found that miR-31 deficiency led to a pronounced decrease in plaque formation (Fig. 3e). The splenomegaly and lymphadenopathy are primarily caused by a large expansion of inflammatory cells^23^. We observed that the conditional knockout of miR-31 in basal keratinocytes dramatically decreased splenomegaly and lymphadenopathy in the IMQ-treated mice (Fig. 3f). Strikingly, we demonstrated that the miR-31-specific ablation in the epidermis resulted in a pronounced decrease in skin thickness in the cKO mice treated with IMQ (Fig. 3g). Moreover, histological analysis of the inflammation revealed a marked decrease in epidermal hyperplasia (acanthosis) and dermal cell infiltration in the cKO mice compared with the miR-31^fl/fl^ controls (Fig. 3h–j). Notably, we detected that the expression of Ki67, a marker strictly associated with cell proliferation, was significantly decreased in the cKO mice when compared with the controls, indicating that the excessive proliferation of basal keratinocytes induced by IMQ was reduced in the absence of miR-31 (Fig. 3k,l). Furthermore, we observed that the miR-31 deletion in the mice treated with IMQ significantly restored the expression of the terminal differentiation markers Keratin 10, Loricrin and Filaggrin close to that of the untreated miR-31^fl/fl^ mice (Fig. 3m). Consistently, we found that miR-31 deletion in the epidermis led to an obvious decrease in plaque formation accompanied with a significant decrease in ear thickness and acanthosis in an IL-23-mediated psoriasis mouse model (Supplementary Fig. 6a–d). However, in addition to IL-1α there was no significant difference of the inflammatory genes between the miR-31^fl/fl^ and cKO mice in both the IMQ-induced and IL-23-mediated mouse models of psoriasis (Supplementary Fig. 7), suggesting a unique role of miR-31 in epidermal hyperplasia. In addition, miR-31 expression in epidermis even decreased in the cKO mice treated with IMQ compared with the untreated cKO controls (Supplementary Fig. 8a), indicating that infiltrated leucocytes did not contribute to the elevated levels of miR-31 in the epidermis. Moreover, the deletion of miR-31 in keratinocytes did not decrease miR-31 expression in splenocytes in the cKO mice treated with IMQ (Supplementary Fig. 8b). Together, these data imply that intrinsic miR-31 plays a pivotal role in keratinocyte proliferation and differentiation, and in epidermal hyperplasia.

### miR-31 directly targets Ppp6c

**Figure 4:**
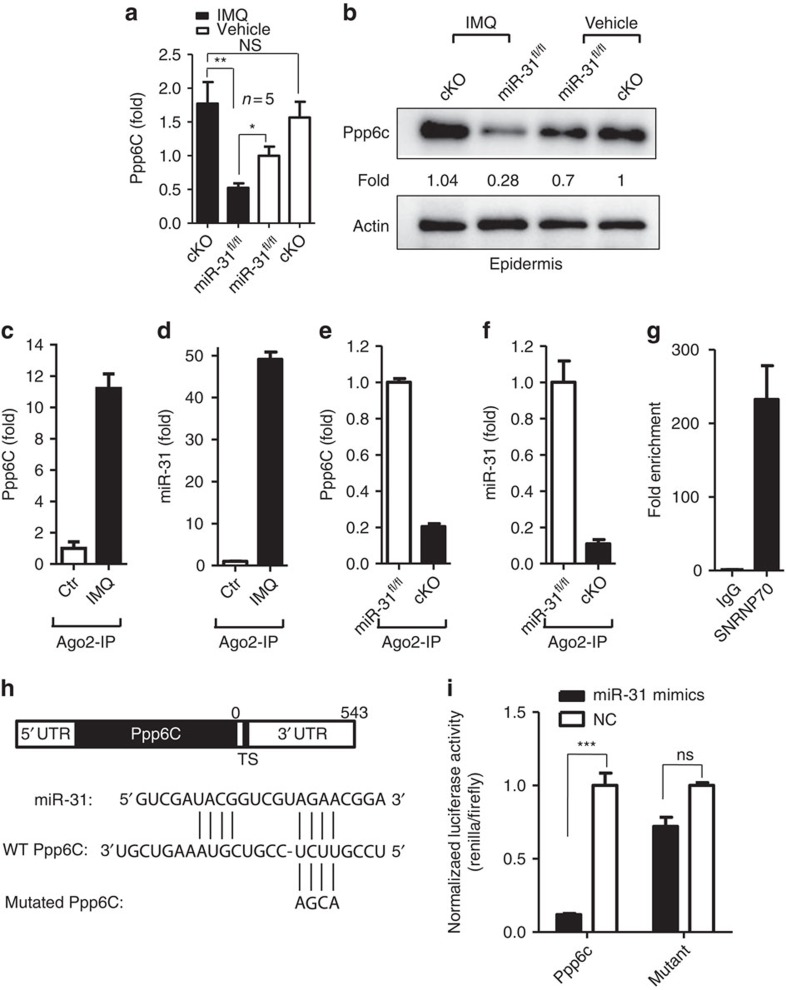
miR-31 directly targets ppp6c. (**a**,**b**) qPCR and western blot analysis of ppp6c expression in the epidermis of cKO and miR-31^fl/fl^ mice treated with IMQ or vehicle. Results (**a**) are presented as the ratio of miRNA to the small nuclear RNA U6, relative to that in vehicle-treated miR-31^fl/fl^ controls. Values (**b**) were expressed as fold changes relative to controls and normalized to β-actin. One representative blot out of four independent experiments is shown. (**c**,**d**) Ago2 was immunoprecipitated from epidermis lysates derived from untreated controls or mice treated with IMQ. Immunoprecipitates were assayed for ppp6c and miR-31. (**e**,**f**) Ago2 was immunoprecipitated from epidermis lysates derived from miR-31^fl/fl^ or cKO mice treated with IMQ. Immunoprecipitates were assayed for ppp6c and miR-31. (**g**) U1 positive control was tested in Ago2 immunoprecipitates from normal epidermis lysates. (**h**) WT and point-mutated 3′ UTR reporter constructs. TS, target site. (**i**) Luciferase activity was determined in NIH3T3 cells that were transfected with miR-31 mimics and the indicated 3′ UTR reporter construct or with the indicated WT or point-mutated 3′ UTR reporter construct (WT UTR or mutant UTR). Results (**c**–**g**) are presented as the ratio of mRNA or miRNA to the β-actin or the small nuclear RNA U6, relative to that in controls. *P<0.05, **P<0.01, ***P<0.001, NS, not significant, two-tailed Student's *t*-test. Data are representative of at least two independent experiments with four to six samples per group in each (mean and s.e.m.).

To identify putative target mRNAs of miR-31, four bioinformatics tools, TargetScan, miRDB, miRWalk and RNA22, were used to predict the potential targets of miR-31 (Supplementary Fig. 9a), and 20 potential target genes were identified (Supplementary Fig. 9b). We compared the mRNA expression profile of the predicted targets in the WT and miR-31^TG^ mice. Only one predicted target of miR-31, ppp6c, was significantly downregulated at the mRNA levels both after the application of IMQ and on the overexpression of miR-31 (Supplementary Fig. 9c). Consistent with our qPCR results, western blot analysis further demonstrated that the ppp6c expression was increased by >3.7-fold in the epidermis of the cKO mice treated with IMQ compared with that of miR-31^fl/fl^ controls. A modest expression of ppp6c in the epidermis of the miR-31^fl/fl^ mice was observed in the absence of IMQ treatment; however, no apparent difference of ppp6c expression was found between the IMQ-treated and untreated cKO mice (Fig. 4a,b). In an IL-23-mediated mouse model of psoriasis, the ppp6c expression was increased more than ∼8.0-fold at the mRNA levels and 12.0-fold at the protein levels in the ear epidermis of the cKO mice injected with IL-23 compared with that of the miR-31^fl/fl^ controls (Supplementary Fig. 10a,b). These data indicate that ppp6c is a potential miR-31 target. In mammalian cells, the majority of mature miRNAs are loaded into Argonaute 2 (Ago2)-associated complexes through which mRNA silencing is conducted^29^. To confirm whether ppp6c is a direct target of miR-31, Ago2 immunoprecipitates of the epidermis derived from untreated and IMQ-treated mice or from miR-31^fl/fl^ and cKO mice treated with IMQ were assayed for ppp6c and miR-31. The levels of ppp6c and miR-31 detected in the psoriatic epidermal immunoprecipitates were 11.2- and 49.1-fold greater, respectively, than those in untreated controls (Fig. 4c,d). Furthermore, we determined that ppp6c and miR-31 expression in the miR-31^fl/fl^ epidermal immunoprecipitates were 5.0- and 9.1-fold greater, respectively, than those in the cKO mice (Fig. 4e,f). Correspondingly, as a technique control we found that a 232.2-fold increase in U1 expression in the Ago2 immunoprecipitates compared with the immunoglobulin-G (IgG) control (Fig. 4g). Unlike ppp6c there was essentially no difference of the expression levels for a non-related miRNA miR-125a in the psoriatic epidermal immunoprecipitates between miR-31^fl/fl^ and cKO mice treated with IMQ (Supplementary Fig. 11a,b). These findings indicate that miR-31 and its target gene ppp6c are specifically associated with Ago2-containing complexes. We next generated a reporter construct that includes the 3′ untranslated region (UTR) of the ppp6c mRNA (Fig. 4h). In contrast to a control construct lacking the target sequence, miR-31 overexpression led to significantly decreased luciferase activity derived from the construct expressing the target sequence, and a 88.12% reduction of the target ppp6c mRNA was achieved (Fig. 4i). Thus, our results demonstrate that miR-31 is capable of directly targeting a sequence within the 3′ UTR of the ppp6c mRNA and that ppp6c is one of the key targets of miR-31 in keratinocytes.

### Ppp6c inhibition is required for miR-31-mediated effects

**Figure 5:**
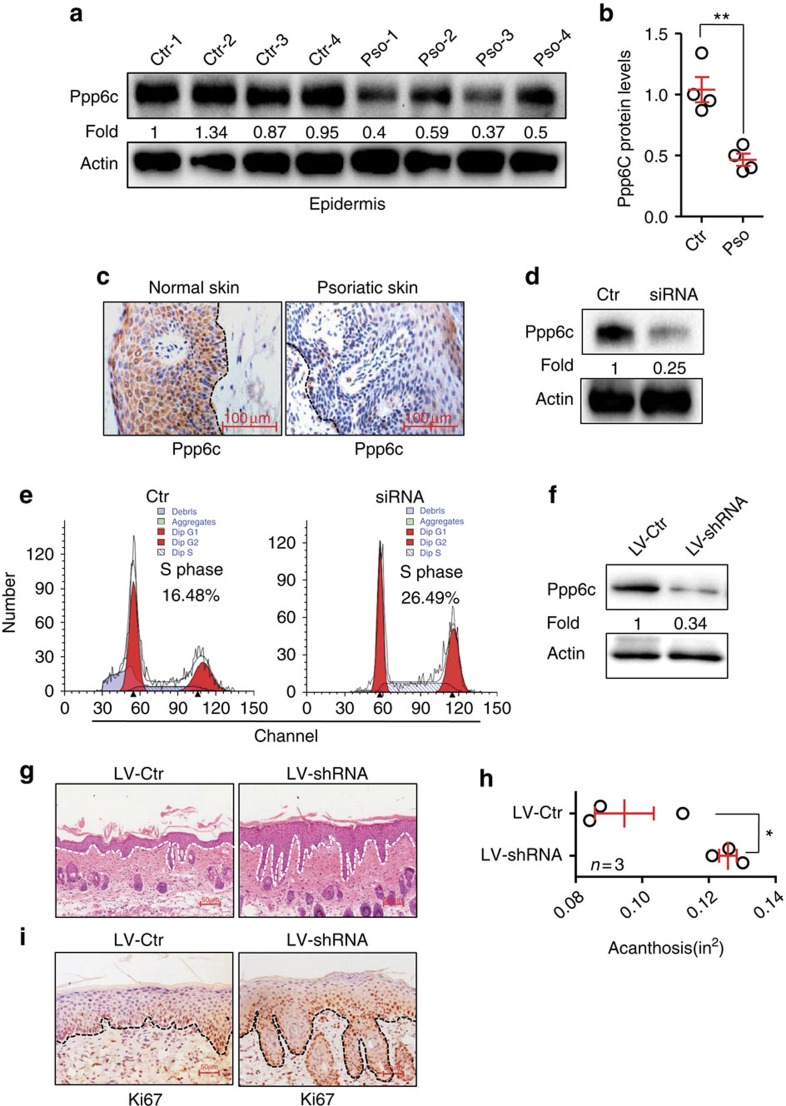
Inhibition of ppp6c is functionally important for the biological effects of miR-31 in epidermal hyperplasia. (**a**,**b**) Western blotting of ppp6c expression in the epidermis of normal skin derived from four healthy individuals (Ctr-1 to 4) or in psoriatic lesions derived from four patients with psoriasis (Pso-1 to 4). (**c**) Immunohistochemical staining of ppp6c in skin sections derived from healthy or psoriatic skin. Scale bar, 100 μm. (**d**) Primary mouse keratinocytes were transfected with scramble siRNA (Ctr) or with ppp6c siRNA (siRNA). Cell lysates were immunoblotted with anti-ppp6c or anti-actin. (**e**) Cell cycle analysis of mouse primary keratinocytes transfected with scrambled siRNA or ppp6c siRNA. (**f**) Western blotting of ppp6c expression in epidermis derived from lentiviral shRNA-control (LV-Ctr) or lentiviral shRNA-ppp6c (LV-shRNA) treated mice. (**g**) H&E staining of the back skin injected with LV-Ctr or LV-shRNA in mice applied with IMQ. Dotted line indicates the border between the epidermis and dermis. Scale bar, 100 μm. (**h**) Acanthosis of the back skin injected with LV-Ctr or LV-shRNA in mice applied with IMQ. (**i**) Immunohistochemical staining of Ki67 in skin sections derived from mice injected with LV-Ctr or LV-shRNA prior to IMQ painting. Dotted line indicates the border between the epidermis and dermis. Scale bar, 50 μm. Values (**a**,**d**,**f**) were expressed as fold changes relative to Ctr-1 (**a**), to scramble siRNA (**d**), or to lentiviral shRNA-control (**f**), and normalized to β-actin. *P<0.05, **P<0.01, two-tailed Student's *t*-test. Data (**c**–**i**) are representative of two independent experiments with three to five samples per group.

We next investigated the ppp6c expression in the epidermis derived from lesional skin of human patients with psoriasis. Of note, we found that the levels of ppp6c protein were significantly decreased in the epidermis of psoriatic lesions compared with the healthy controls (Fig. 5a,b). In contrast to normal skin, a diminished expression of ppp6c was revealed in the psoriatic lesions by immunohistochemistry staining (Fig. 5c). To explore the biological function of ppp6c, we depleted ppp6c in keratinocytes with specific siRNA (Fig. 5d). Strikingly, in contrast to the controls, the dampening of ppp6c expression in keratinocytes resulted in an increased percentage of cells in S phase (Fig. 5e). However, silencing ppp6c did not alter the miR-31 expression in NHEK (Supplementary Fig. 11c). To silence ppp6c *in vivo*, we generated a lentivirus that expressed green fluorescent protein and ppp6c short hairpin RNA (shRNA) driven by the CMV immediate early promoter and the RNA Pol III-dependent, human U6 promoter (U6), respectively^30^. Lentiviral shRNA-ppp6c downregulated ppp6c protein in epidermis *in vivo* (Fig. 5f). Moreover, ppp6c silencing led to an increase in the epidermis thickness and an enhanced proliferation of keratinocytes as reflected by the elevated levels of Ki67 (Fig. 5g–i). These data suggest that ppp6c is possibly functionally important in the regulation of epidermal hyperplasia in psoriasis.

### NF-κB signalling inhibits Ppp6c expression mediated by miR-31

**Figure 6:**
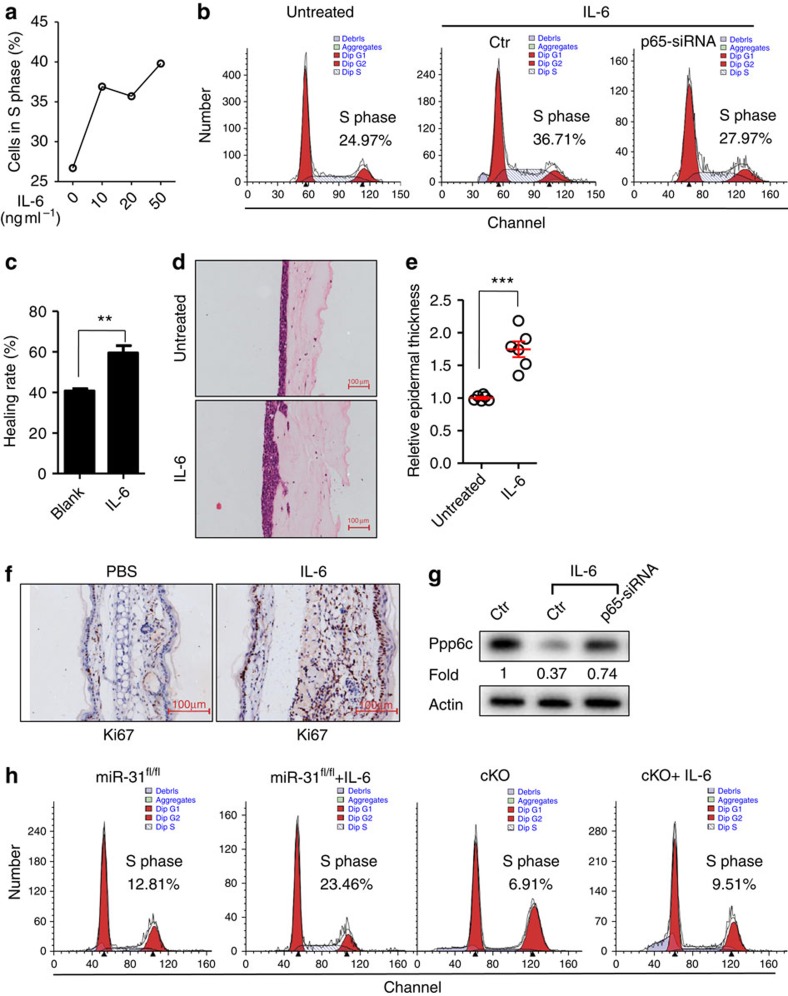
NF-κB signaling inhibits ppp6c expression by inducing miR-31. (**a**) Undifferentiated NHEK proliferation induced by various concentrations of IL-6 for 24 h, and analysed by BrdU incorporation assay. (**b**) NHEK were transfected with scramble siRNA (Ctr) or with p65 siRNA (p65 siRNA). Cell cycle analysis of NHEK or transfected NHEK treated without or with IL-6 for 24 h. (**c**) *In vitro* wound healing rate of NHEK treated without or with IL-6 for 16 h. (**d**,**e**) Three-dimensional organotypic culture of HaCaT keratinocytes treated without or with IL-6. Scale bar, 100 μm. (**f**) 1 μg recombinant mouse IL-6 (in 25 μl PBS) or PBS was injected i.d. in ears of C57BL/6J mice. Ear sections were prepared for Ki67 staining 3 days after IL-6 administration. Scale bar, 100 μm. (**g**) NHEK were transfected with scramble siRNA (Ctr) or with p65 siRNA (p65 siRNA). Western blotting of ppp6c expression in NHEK with or without IL-6 treatment for 24 h. (**h**) Cell cycle analysis of primary mouse keratinocytes derived from miR-31^fl/fl^ or cKO mice in absence or presence of IL-6. Values were expressed as fold changes relative to non-stimulated HaCaT keratinocytes (**e**) or to non-stimulated NHEK (**g**) and normalized to β-actin. IL-6 was used at the concentration of 50 ng ml^−1^ (**b**–**e**,**g**,**h**). **P<0.01, ***P<0.001, two-tailed Student's *t*-test. Data are representative of at least two independent experiments.

Given that NF-κB is a key regulatory component in cellular differentiation and proliferation, we next examined whether NF-κB activation modulates ppp6c expression in proliferating keratinocytes. We stimulated NHEK with various concentrations of IL-6, and assessed the proliferation of NHEK by BrdU staining. Consistent with a published report^31^, IL-6 strongly induced NHEK proliferation (Fig. 6a), which was abolished by the siRNA-mediated knockdown of p65 in cultures (Fig. 6b). We further observed that the addition of IL-6 caused a significant increase in the healing rate of NHEK by 16 h following sterile micropipette injury compared with the controls (Fig. 6c). Three-dimensional organotypic cultures confirmed that IL-6 stimulation remarkably enhanced keratinocyte proliferation (Fig. 6d,e). Moreover, injection of recombinant IL-6 into ears resulted in an increased expression of Ki67 in mouse epidermis (Fig. 6f). Thus, our data suggest that NF-κB signalling triggered by IL-6 is critical for NHEK proliferation.

To address whether NF-κB signalling modulates ppp6c expression, we measured the ppp6c protein levels in NHEK stimulated with IL-6. The addition of IL-6 in NHEK led to a diminished expression of ppp6c at the protein levels, and its expression was rescued by siRNA-mediated knockdown of p65 (Fig. 6g). Of note, we detected a dramatic increase of proliferation in the miR-31^fl/fl^ keratinocytes, but not the cKO keratinocytes when stimulated with IL-6, indicating that IL-6 is not able to trigger keratinocyte proliferation in the absence of miR-31 (Fig. 6h). Similarly, we found that the addition of IL-17A in cultured keratinocytes caused a decreased expression of ppp6c at the protein levels (Supplementary Fig. 12a). Moreover, an *in vivo* study demonstrated there was a significant decrease of ppp6c in the epidermis of lesional skin derived from IL-17A^+/+^ mice compared with IL-17A^−/−^ mice when both were treated with IMQ for 7 days (Supplementary Fig. 12b,c). Together, these experiments suggest that NF-κB activation triggered by inflammatory cytokines inhibits ppp6c expression mediated by miR-31 induction in keratinocytes.

### Anti-miR-31 administration decreases epidermal hyperplasia

**Figure 7:**
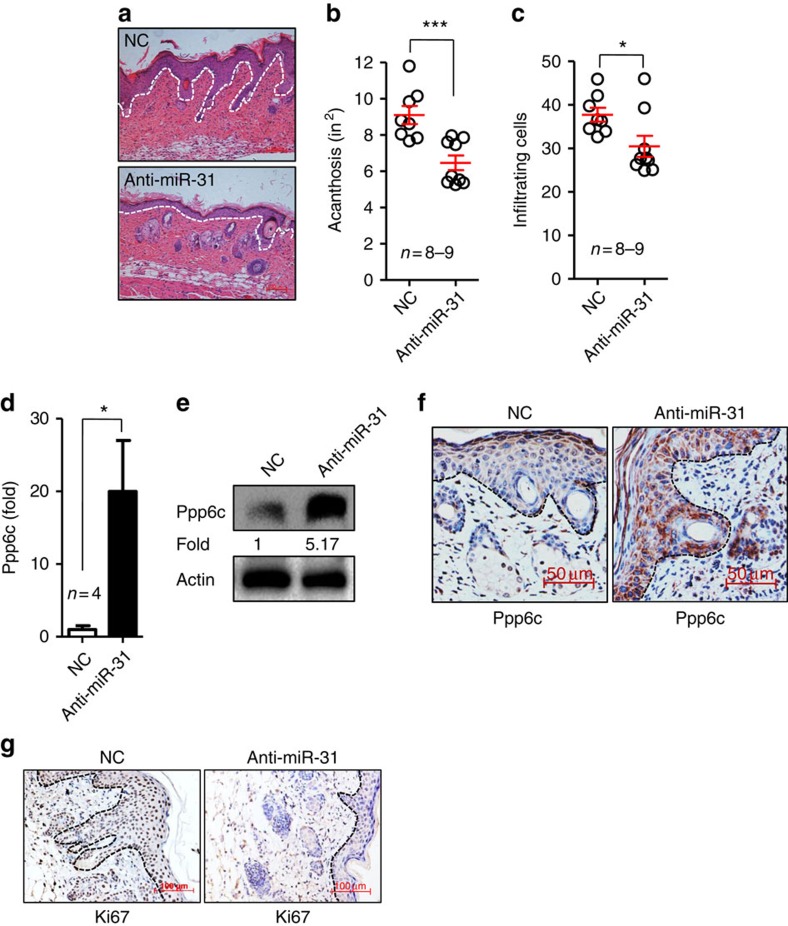
Administration of antagomir-31 decreases epidermal hyperplasia and dermal cellular infiltration induced by IMQ. Mice were injected subcutaneously with an irrelevant antagomir (NC) or an antagomir to miR-31 (anti-miR-31). The first injection was administered 3 days before the application of IMQ and thereafter was performed every other day until the end of the experiment. (**a**) H&E staining of the back skin derived from mice injected with NC (upper panel) or anti-miR-31 (lower panel). Scale bar, 100 μm. (**b**,**c**) Acanthosis and dermal cellular infiltrates were quantitated for mice treated with NC or anti-miR-31. (**d**,**e**) Ppp6c mRNA and protein levels in NC- or anti-miR-31-treated mice. (**f**,**g**) Immunohistochemical staining of ppp6c or Ki67 in skin sections derived from NC- or anti-miR-31-treated mice after induction of skin phenotype by IMQ (*n*=8–9). Scale bar, 50 μm (**f**) or 100 μm (**g**). For all measurements (**c**), the median number of specifically stained dermal nucleated cells was counted in three high-power fields per section. Results (**d**) are presented as the ratio of mRNA to the β-actin, relative to that in NC-treated mice. *P<0.05, ***P<0.001, two-tailed Student's *t*-test. Data (**a**–**g**) are representative of at least two independent experiments with four to nine samples per group in each (mean and s.e.m.).

Antagomirs are modified antisense oligonucleotides, and exhibit superior miRNA-inhibiting properties when applied in mice^32^. We administered antagomirs to block the miR-31 seed sequence (anti-miR-31) and to test the inhibitory effects of anti-miR-31 on the disease development in the IMQ-induced psoriasis mouse model. As expected, there was a pronounced decrease in both acanthosis and dermal cellular infiltration after the anti-miR-31 treatment (Fig. 7a–c). Correspondingly, we found enhanced ppp6c mRNA and protein levels in the epidermis after the administration of anti-miR-31 (Fig. 7d,e). The increased ppp6c expression after the anti-miR-31 treatment in epidermis was further confirmed by immunohistochemistry analysis (Fig. 7f). Moreover, the anti-miR-31 administration markedly reduced the keratinocyte hyperproliferation as indicated by the Ki67 levels (Fig. 7g). Thus, our data again indicate that miR-31 and its target ppp6c are critical factors in epidermal hyperplasia in psoriasis.

## Discussion

Increased NF-κB activation was demonstrated in lesional psoriatic skin compared with non-lesional psoriatic skin^7^. Major advances highlighting important roles for NF-κB in the aetiology of psoriasis have been achieved^33^. In contrast, very little is known about the intrinsic factor(s) induced by NF-κB activation in keratinocytes in the promotion of epidermal hyperplasia. In this study, we demonstrate that inflammatory cytokines activate NF-κB signalling and induce miR-31, which represses ppp6c, a negative regulator of the cell cycle, thereby contributing to basal keratinocyte proliferation and epidermal hyperplasia. Our data provide the mechanistic evidence for miRNA-mediated regulation involved in the hyperproliferative keratinocytes in psoriasis. These findings also suggest potential therapeutic targets for psoriasis treatment.

Although the initial events triggering a psoriatic lesion are still not defined, a complex interplay between dysfunctional keratinocytes and abnormal activation of the innate and the adaptive immune system may drive epidermal hyperproliferation and aberrant keratinocyte differentiation in psoriasis. Epidermal keratinocytes are responsive to dendritic cell-derived and T-cell-derived cytokines such as IFNs, TNF, IL-6, IL-17 and the IL-20 family of cytokines and *vice versa*, they are able to release proinflammatory cytokines and chemokines to sustain or even amplify the chronic inflammatory disease loop in lesional skin in psoriasis^1^. Using the ChIP assay, the siRNA-mediated knockdown of NF-κB subunit p65 or luciferase reporter constructs driven by the specific promoter, we demonstrate that inflammatory cytokines are capable of triggering miR-31 transcription directly or indirectly through NF-κB signalling, which plays an essential role both in cell cycle regulation and inflammatory response, and critically connects keratinocytes with lymphocytes in the pathogenesis of psoriasis^4^. Thus, the overexpression of miR-31 in psoriatic keratinocytes, likely as a consequence of the production of excess inflammatory cytokines in both skin lesion and plasma of patients, may therefore contribute to the epidermal hyperplasia that occurs in psoriasis. In our study NF-κB acts as an upstream enhancer of miR-31 in keratinocytes, while another report shows that miR-31 targets serine/threonine-protein kinase 40 (STK40), a negative regulator of the NF-κB signalling pathway^21^. These findings that suggest different target genes of miR-31 in fact indicate that the miR-31-mediated positive feedback loop may amplify NF-κB activity to pathological levels in epidermal hyperplasia.

miR-31 is universally expressed in a variety of tissues, and has been shown to negatively regulate lymphatic vascular lineage-specific differentiation^34^, to sensitize breast cells to apoptosis by targeting protein kinase c-ɛ^35^, to enhance vascular smooth muscle cell proliferation via inhibiting its target gene, the large tumor suppressor homologue 2 (ref. 36), to regulate keratinocyte differentiation with a targeting effect on factor-inhibiting hypoxia-inducible factor 1 (ref. 37) and to control hair cycle-associated genes in mouse skin^38^. To our knowledge, no prior studies have addressed the possible direct intrinsic role of miR-31 in keratinocyte proliferation or differentiation and in the pathogenesis of psoriasis. Here we demonstrate that the conditional knockout of miR-31 leads to decreased keratinocyte hyperproliferation mediated by NF-κB signalling, prevents Ki67 expression, inhibits acanthosis and reduces the disease severity in two psoriasis mouse models. Our data identify miR-31 as a downstream target of NF-κB and highlight the critical role of NF-κB-mediated post-transcriptional regulation for epidermal hyperplasia in psoriasis. The critical role of miR-31 in hyperproliferative keratinocytes is further indicated by the observation that repeated intradermal (i.d.) injection of anti-miR-31 in the IMQ-induced mouse model results in a significant improvement of the psoriasiform phenotype.

Although miR-31 was reported to modulate inflammatory cytokine and chemokine expression in keratinocytes by suppressing STK40 (ref. 21), the *in vivo* function of miR-31 and the underlying mechanism by which it regulates cell proliferation and differentiation in psoriasis has been poorly explored. Here we identify that miR-31 induced by NF-κB activation directly targets ppp6c to promote keratinocyte hyperproliferation. We present several lines of evidence to support that ppp6c is one of the primary targets for miR-31 in keratinocytes. First, four separate bioinformatics tools predict that miR-31 targets a sequence in the 3′ UTR of ppp6c mRNA. Second, ppp6c expression is significantly decreased in diseased epidermis of miR-31^fl/fl^ control mice but not in the epidermis of cKO mice treated with IMQ or IL-23. Third, ppp6c and miR-31 expression in Ago2 immunoprecipitates of lesional epidermis derived from miR-31^fl/fl^ animals is markedly enriched compared with that in immunoprecipitates from cKO mice, suggesting that ppp6c and miR-31 associate within Ago2, the effector element of the miRNA-induced silencing complex, which directly binds to miRNAs and subsequently mediates mRNA repression^39^. Fourth, miR-31 overexpression in NIH3T3 cells results in significantly decreased luciferase activity after the transfection of the cells with the construct expressing the target sequence in the 3′ UTR of ppp6c. Fifth, the administration of anti-miR-31 blocks miR-31 function, and enhances the ppp6c mRNA and protein levels *in vivo*.

A marked increase in the percentage of normally quiescent basal keratinocytes during cell cycle progression is one of the essential characteristics of epidermal hyperproliferation in psoriasis^40^. However, post-transcriptional evidence by which NF-κB regulates the aberrant cell cycle of epidermal basal keratinocytes is lacking. The data presented here show that NF-κB induces miR-31, which directly inhibits ppp6c, thereby increasing basal keratinocyte proliferation. Ppp6c has been shown to regulate mitotic spindle formation by dephosphorylating Aurora A bound to its activator TPX2 (ref. 41). The role of ppp6c in keratinocyte hyperproliferation and in the pathogenesis of psoriasis is not known. We showed that ppp6c expression is diminished in the epidermis of lesional skin from patients with psoriasis, and that knockdown of ppp6c by siRNA or shRNA results in an enhanced percentage of proliferating keratinocytes *in vitro* and promotes psoriasiform skin disease in the IMQ-induced mouse model. These data noted above indicate that modulation of ppp6c expression in keratinocytes may provide a basis for studies that lead to potential therapies for psoriasis.

In conclusion, the present study has shown that the activation of NF-κB signalling triggered by inflammatory cytokines induces miR-31 expression in keratinocytes. The conditional deletion of miR-31 decreases epidermal hyperplasia and attenuates psoriasiform phenotype in mouse models of psoriasis. miR-31 directly targets ppp6c, an essential element regulating cell cycle, which has been shown to be diminished in the epidermis of lesional skin from patients with psoriasis. Our findings reveal a previously unknown post-transcriptional mechanism for NF-κB-mediated hyperproliferative keratinocytes and highlights a critical role of the NF-κB–ppp6c axis in epidermal hyperplasia of psoriasis.

## Methods

### Mice

The Keratin 5-Cre transgenic mice were kindly provided by Dr Xiao Yang (State Key Laboratory of Proteomics, Genetic Laboratory of Development and Disease, Institute of Biotechnology, Beijing, China)^42^. IL-17A^−/−^ mice were kindly provided by Dr Yoichiro Iwakura (Research Institute for Biomedical Sciences, Tokyo University of Science, Tokyo, Japan)^43^. NF-κB luciferase reporter mice were kindly provided by Dr Jiong Deng (Shanghai Jiao Tong University School of Medicine, Shanghai, China)^44^. Age- and sex-matched mice at 8–12 weeks of age were used for all experiments. All mice were kept under specific pathogen-free (SPF) conditions in compliance with the National Institutes of Health Guide for the Care and Use of Laboratory Animals with the approval (SYXK-2003-0026) of the Scientific Investigation Board of Shanghai Jiao Tong University School of Medicine, Shanghai, China. To ameliorate any suffering of mice observed throughout these experimental studies, mice were euthanized by CO_2_ inhalation.

### IMQ-induced mouse model of psoriasis

Female C57BL/6J mice (8–12 weeks of age) purchased from Shanghai SLAC Laboratory Animal Co. (Shanghai, China) were maintained under SPF conditions. The mice were applied to a daily topical dose of 62.5 mg of IMQ cream (5%) (MedShine, #120503; China) on the shaved back for 7 consecutive days. At the indicated days, skin thickness was measured using a micrometer (Mitutoyo). Control mice were treated with a same dose of vehicle cream. All procedures were approved and supervised by Shanghai Jiao Tong University School of Medicine Animal Care and Use Committee.

### RNA reverse transcription and qPCR

Total RNA was extracted from keratinocytes and skin biopsies using the Trizol reagent (Invitrogen, #15596-026) and NanoDrop spectrophotometer (ND-1000) was used for RNA quality control. For epidermis RNA extraction, an overnight incubation of skin samples at 4 °C in dispase II (2.5 U ml^−1^) (BD, #354235) was performed to completely separate the epidermis from the dermis. Complementary DNA was synthesized using SuperScript First-Strand Synthesis System (Invitrogen, #1209992). qPCR was carried out with the FastStart Universal SYBR Green Master (Roche, #04913914001) in a ViiA 7 Real-Time PCR system (Applied Biosystems). The relative expression of target genes was confirmed using quantity of target gene/quantity of β-actin. Primers sequences were used as follows: Unc93b1, forward primer, 5′-CACCCTTACTTACGGCGTCTA-3′, reverse primer 5′-CATGTTGCCATACTTCACCTCT-3′; Jph4, forward primer, 5′-CAGCAGACGCCCTCCTAAAAG-3′, reverse primer, 5′-GGGTTGTAGATCCTGGGCTATC-3′; Xpnpep3, forward primer, 5′-GCCGACATCTTAGCCTACCC-3′, reverse primer, 5′-TATTTTCGAGCAGCCTGCCA-3′; Narg1, forward primer, 5′-TCAAAGCAGCGAGAATTGCTA-3′, reverse primer, 5′-TGGGGCCTCCAATTTCTTCG-3′; Fbxw11, forward primer, 5′-TACCAGAGCAAGGCTTAGATCA-3′, reverse primer, 5′-TTCTTTCTGAGAGTCCCTTCCA-3′; Nup50, forward primer, 5′-AACAGAGCCGTAAAGAAGGCA-3′, reverse primer, 5′-GCCACCAAATCCAGAAAACCC-3′; Irf4, forward primer, 5′-CCGACAGTGGTTGATCGACC-3′, reverse primer, 5′-CCTCACGATTGTAGTCCTGCTT-3′; Slc25a28, forward primer, 5′-GGGCTGAACGTCACAGCAA-3′, reverse primer, 5′-TGCACCATTGGCAATATGGCT-3′; Nipsnap3, forward primer, 5′-TCAGGTGTGCTCGCCTTTC-3′, reverse primer, 5′-CCTACAGTCCAATATCCCACCA-3′; Hip2, forward primer, 5′-CAGAACCAGATGACCCCCAAG-3′, reverse primer, 5′-CGAGCTGTCTGCTTGAACATT-3′; Gabrp, forward primer, 5′-CAGACCCACGGCTAGTGTTC-3′, reverse primer, 5′-AGAGGCGGATGAGCCTGTT-3′; Pwp1, forward primer, 5′-GAGTCTCTGTTGGGTCTTACGG-3′, reverse primer, 5′-GGTTGTCAGTCGGCTTAATCAAG-3′; Topbp1, forward primer, 5′-CAGGATTGTTGGTCCTCAAGTG-3′, reverse primer, 5′-ACAGGATACAGTTACGTCAGACA-3′; Ccr2, forward primer, 5′-ATCCACGGCATACTATCAACATC-3′, reverse primer, 5′-CAAGGCTCACCATCATCGTAG-3′; Stx12, forward primer, 5′-ATGTCCTACGGTCCCTTAGAC-3′, reverse primer, 5′-GGCTGATCCGCTGGATGTT-3′; Sugt1, forward primer, 5′-CCGTGATGGTATTGCCGATGT-3′, reverse primer, 5′-GGCAGAAGCGTAGTCTTTTTCA-3′; Ppp6c, forward primer, 5′-CCGCTGGATCTGGACAAGTAT-3′, reverse primer, 5′-ACACTGGCTGAACATTCGACT-3′; Lin7c, forward primer, 5′-GGCCTCAAACGAGGAGATCAG-3′, reverse primer, 5′-AGCTCTACCGCTTTCTCATGG-3′; Fermt1, forward primer, 5′-TTTCGGCTGTGGTGTTTAAGG-3′, reverse primer, 5′-GGATGTCTTCAATCACTGGCTC-3′; Ppl, forward primer, 5′-GGCAGTGAAAGAGGCCGAC-3′, reverse primer, 5′-GGCTATCCACCAGAGGAAGGT-3′. Keratin 10, forward primer,5′-GCCTCCTA CATGGACAAAGTC-3′, reverse primer, 5′-GCTTCTCGTACCACTCCTTGA-3′; Involucrin, forward primer, 5′-TCCTCCAGTCAATACCCATCAG-3′, reverse primer, 5′-CAGCAGTCATGTGCTTTTCCT-3′; Filaggrin, forward primer,5′-CAGCTGACAGGCAAGGGC-3′, reverse primer, 5′-CTGTGAGCTCCTACTGCCTG-3′. To measure mature miR-31 levels, 50 ng of total RNA was reverse transcribed using the TaqMan miRNA reverse transcription kit, miR-31RT primers and U6 snRNA (Applied Biosystems). The complementary DNAs were then analysed by qPCR using the TaqMan probes for miR-31 and U6 snRNA. Quantification of relative miRNA expression was measured by the comparative CT (critical threshold) method, normalized to endogenous U6 expression and determined by the formula 2^−ΔΔCT^.

### *In situ* hybridization

*In situ* hybridization was performed on 10-μm-thick frozen sections of IMQ-induced mouse psoriasiform specimens and control specimens. Briefly, after incubated in acetylation solution (0.02 M HCl, 1.3% trietanolamin and 0.25% acetic anhydride in diethyl pyrocarbonate-treated water) for 10 min at room temperature (RT), sections were treated with proteinase K (5 μg ml^−1^) in PBS for 10 min, washed and prehybridized for 6 h. Hybridization with mmu-miR-31 5′-DIG and 3′-DIG-labelled miRCURY LNA detection probe (Exiqon, #39153-15) was performed overnight at 50 °C. Slides were then washed with 5 × SSC buffer followed by incubation in 0.2 × SSC buffer for 1 h at 60 °C. The probe binding was detected by incubating sections with anti-DIG-alkaline phosphatase antibody (Ab) (1:2,000, Roche, #11093274910) for overnight at 4 °C. Sections were visualized by using NBT/BCIP ready-to-use tablets (Roche, #11697471001), according to the manufacturer's instructions.

### Human subjects

Psoriatic skin samples were obtained by punch biopsy under local lidocaine anaesthesia. Normal adult human skin specimens were taken from healthy adults undergoing plastic surgery. The fresh tissue samples were snap frozen in liquid nitrogen and stored at −80 °C. All individuals provided informed consent. The study was performed in accordance with the declaration of Helsinki Principles and approved by the Research Ethics Board of the Second Affiliated Hospital of Xi'an Jiaotong University, Xi'an, China.

### *In vivo* activation of NF-κB assay

NF-κB luciferase reporter mice with C57BL/6J background in which luciferase expression is driven by the NF-κB-dependent portion of the HIV-1 long terminal repeat^44^ were treated with IMQ. For bioluminescence imaging, mice were given 150 μg g^−1^ of D-luciferin in PBS by i.p. injection. Ten minutes after injection, bioluminescence imaging was captured with a charge-coupled device camera (IVIS; Xenogen).

### Immunofluorescence staining

NHEKs were seeded on glass coverslips, cultured for 36 h and stimulated with IL-6 for 1 h. Cultured cells were washed three times in PBS and fixed for 15 min at RT, and incubated in Blocking Buffer (1 × PBS/5% BSA/0.3% Triton X-100) for 1 h. Anti-p65 rabbit Ab (Cell Signaling Technology, #D14E12) was diluted at 1:50 in Ab Dilution Buffer (1 × PBS/1% BSA/0.3% Triton X-100). The cells were stained with anti-p65 rabbit Ab and incubated for overnight at 4 °C. Thereafter, the cells were rinsed three times in PBS and incubated in fluorochrome-conjugated secondary Ab with a 1:800 dilution for 1 h at RT in the dark. After being washed in PBS and air drying, the cover lips were mounted with Prolong Gold Anti-Fade Reagent with DAPI (Invitrogen, #P-36931). Image analysis was performed using a fluorescent microscope in combination with Axio Cam HRc (ZEISS, Germany) and AxioVision Rel 4.7 analysis software.

### Flow cytometric analysis

Single-cell suspensions from mouse IMQ-induced psoriasiform skin or normal skin were prepared in PBS. Cells were fixed with 10 volumes of pre-warmed Lyse/Fix Buffer (BD, #51–2090KZ) by vortexing before incubation at 37 °C for 10 min. Cells were then washed and permeabilized with 1 ml of pre-chilled Perm buffer III (BD, #558050) for 30 min on ice, followed by Fc blocking Ab (BioLegend, #101301) treatment. Anti-phosphorylated-p65 rabbit Ab (Cell Signaling Technology, #3033) were used to stain the cells for 60 min at RT. Thereafter, cells were stained with PE-anti-CD45 Ab (BioLegend, #103106) and Alexa Fluor 488-anti-rabbit IgG Ab (Life Technologies, #A11034) for 30 min at 4 °C protected from light. Finally, cells were washed, resuspended and analysed with a BD FACSCanto II Flow Cytometer.

### Keratinocyte isolation and culture

Newborn mouse back skin was incubated in dispase II for overnight at 4 °C. The epidermis was gently scraped and washed using cold HBSS (GIBCO, #14175-095). The epidermal cells were separated following incubation with 5 ml 0.05% Trypsin (GIBCO, #25200-056) at 37 °C for 10 min, and subsequently the digestion was neutralized with 10 ml cold TNS (HBSS containing 5% FBS chelated with Chelex 100 Resin; #143–2832, Bio-Rad). After centrifugation at 500 r.p.m. for 10 min, 10 ml Medium 154CF (GIBCO, #M-154CF-500) was used to re-suspend the pellet. Cells were seeded in 24-well plate, which was coated with 0.5 ml coating matrix buffer before using. Primary murine keratinocytes were grown in medium 154CF containing 0.05 mM CaCl_2_, HKGS (GIBCO, #S-001-5) and Pen Strep/Fungizone (Invitrogen, #15240-096) at 37 °C, 5% CO_2_. Normal human epidermal keratinocytes (NHEK, Lifeline Cell Technology, #FC-0007) stored in liquid nitrogen were defrosted and cultured in serum-free basal medium with growth factors (Lifeline Cell Technology, #LL-0007). Medium was refreshed every 2 days and cells were subcultured according to the cell fusion. Cells at passage 2–6 were used for subsequent experiments.

### ChIP assay

ChIP assays were performed using the SimpleCHIP enzymatic chromatin immunoprecipitation kit (Cell Signaling Technology, #9002) according to the manufacturer's protocol with minor modifications. In brief, cells were harvested and cross-linked with 1% (v/v) formaldehyde for 10 min at RT. Subsequently, nuclei were isolated by the lysis of cytoplasmic fraction, and chromatin was digested into fragments of 150–900 bp by micrococcalnuclease (400 gel units) for 20 min at 37 °C, followed by ultrasonic disruption of the nuclear membrane using a standard microtip and a Branson W250D Sonifier (four pulses, 60% amplitude and duty cycle 40%). The sonicated nuclear fractions were divided for input control and for overnight incubated at 4 °C with 5 μg of either anti-p65 Ab or the negative control IgG. After incubation with 30 μl of ChIP grade protein G-agarose beads for 2 h at 4 °C, the Ab-protein-DNA complexes were then eluted from the beads and digested by Proteinase K (40 μg) for 2 h at 65 °C, followed by spin column-based purification of the DNA. Finally, genomic DNA recovered from the ChIP assays were qPCR amplified with primers specific to the p65-binding elements of the miR-31 promoter region. The primers used for detection of miR-31 promoter sequence were as follows: forward, 5′-GCAGTGGAAAGGTTCAGTGC-3′ and reverse, 5′-TATCCTCAACCCTCCGTGTC-3′. The specificity of each primer set was verified by analysing the dissociation curve of each gene-specific PCR product.

### RNA interference

Custom and chemical modified siRNA was designed to target mouse ppp6c (GenePharma). siRNA-210, 5′-AGUCGAAUGUUCAGCCAGUTT-3′, 3′-ACUGGCUGAACAUUCGACUTT-5′; siRNA-410, 5′-GACCGUAUUACACUUUUAATT-3′, 3′-UUAAAAGUGUAAUACGGUCTT-5′; siRNA-586, 5′-GGCGGUUUAUCUCCUGAUATT-3′, 3′-UAUCAGGAGAUAAACCGCCTT-5′. Modified siRNA was also designed to target mouse p65. siRNA-1822, 5′-GGAGUACCCUGAAGCUAUATT-3′, 3′-TTCCUCAUGGGACUUCGAUAU -5′; siRNA-1254, 5′-GGACCUAUGAGACCUUCAATT-3′, 3′-TTCCUGGAUACUCUGGAAGUU-5′; siRNA-396, 5′-GGCCUUAUGUGGAGAUCAUTT-3′, 3′-TTCCGGAAUACACCUCUAGUA -5′. Non-specific siRNA duplex served as a control (GenePharma). To knock down ppp6c or p65 *in vitro*, primary murine keratinocytes were transfected with siRNA-410 (ppp6c, 45 pmol ml^−1^) or with siRNA-396 (p65, 45 pmol ml^−1^) by RNAiMAX Reagent (Invitrogen, #13778).

### Western blotting

Cultured keratinocytes or epidermis sheets were lysed in radioimmunoprecipitation assay buffer supplemented with protease and phosphatase inhibitor cocktail (Thermo Scientific, #78440). Mouse anti-actin Ab (1:3,000; Cell Signaling Technology, #3700S), rabbit anti-ppp6c Ab (1:1,000; Abcam, #EPR8764), rat anti-p65 Ab (Cell Signaling Technology, #8242S) and rabbit anti-mouse Ki67 Ab (eBioscience, #42–5698) were used. Data was analysed using Image Lab software (Bio-Rad). Images have been cropped for presentation. Full size images are presented in Supplementary Fig. 13.

### Luciferase reporter assays

For promoter reporter assay, pGL3-basic vector (Promega, #E1751) was used to clone the promoter of miR-31. Site-specific mutant was generated by PCR. The following primers were used: forward primer, 5′-CCGCTCGAGCGGCTGCGAGAAGCTCCCACCCG-3′; reverse primer, 5′-CCCAAGCTTGGGGGCCCCGAAGCCTCCTCAAC-3′; site-specific mutant, 5′-GGGCGAAGGTGCTTTCACGGTTACTCCCGCGCGC-3′. HaCaT keratinocytes were seeded in a 24-well plate with a density of 5 × 10^4^ per well one day before transfection and then each well was transfected with a mixture of 500 ng pGL3 luciferase vector and 50 ng pRL-TK renilla vector using Lipofectamine 2000 Transfection Reagent (Invitrogen, #11668-019). Twenty-four hours post transfection, cells were starved by removing serum from the medium for 24 h. Then cells were treated with inflammatory cytokines (50 ng ml^−1^) for another 24 h before being lysed, and luciferase activity was measured on a microplate reader (Berthold, TriStar LB941) by using the Dual-Luciferase Reporter Assay System (Promega, #E1910). The ratio of firefly luciferase to renilla luciferase was calculated for each well.

For 3′ UTR reporter assay, 3′ UTR fragments of ppp6c were cloned into psiCHECK-2 vector (Promega, #C8021). Site-specific mutant was generated by PCR. The following primers were used: Ppp6c, forward primer, 5′-AATCTCGAGGCCTTTGCCATCCCACT-3′; reverse primer, 5′-TCGGCGGCCGCATTATCATCCAACAAAGGACAGTGA-3′; site-specific mutant, 5′-GCGGCGGCCGCGTTAAAAAAAAAGATGCTAGGCAGCATTTC-3′. NIH3T3 cells were grown in DMEM (Thermo Scientific, #SH30243.01B) supplemented with heat-inactivated 10% FBS (Gibco, #10437036) in an atmosphere of 5% CO_2_ at 37 °C. Cells with 60% confluence were co-transfected with 100 ng 3′-UTR luciferase reporter vector and 50 pmol miR-31 mimics (GenePharma) using TurboFect (Thermo Scientific, #R0531). Twenty-four hours post transfection, the luciferase activity was measured using Dual-Luciferase Reporter Assay System.

### Generation of miR-31^fl/fl^/Keratin 5-Cre mice

The miR-31 locus (mmu-mir31 ENSMUSG00000065408, http://www.ensembl.org/index.html) is on chromosome 4 (*Mus musculus*) and encodes the miR-31. To create loxP-miR-31-loxP mice, a targeting vector was designed to insert with an frt-flanked PGK-neo cassette and a loxP site upstream of miR-31 and a second loxP site downstream of miR-31. LoxP site was a 34 bp length DNA sequence that can be recognized by Cre recombinase catalyses. If two loxP sites are introduced in the same orientation into a genomic locus, expression of Cre results in the deletion of the loxP-flanked DNA sequence. After linearization, the vector was electroporated into 129S6 derived embryonic stem (ES) cells. The harvested ES cells were screened with 300 μg ml^−1^ G418 and 2 μM Gan C for 8 days and ascertained by PCR. The ES cells with right homologous recombination were injected into blastocyst. After birth, the chimeric mice were bred with 129S mice to generate the heterozygotes. The resulting loxP-miR-31-loxP mice were backcrossed onto C57BL/6J background for five generations and bred with Keratin 5-Cre transgenic mice. P1 and P2 were used to genotype the miR-31 floxed allele (1,064 bp) and the miR-31 deleted allele (235 bp). P1, 5′-GGGAGGATTGGGAAGACAAT-3′; P2, 5′-TGAGGACTTGCAAACGTCAG-3′. Excision by Keratin 5-Cre was complete for all pups used in experiments. miR-31^fl/fl^ and cKO mice did not develop any skin phenotypes at basal conditions on either strain of mice at least for 6 months.

### Generation of miR-31^TG^ mice

To generate miR-31^TG^ mice, the genomic DNA fragment with a size of about 500 bp corresponding to pri-mmu-miR-31 was amplified from mouse genomic DNA with a primer pair of 5′-TCTAGAGGCTGTATTCATCGTTGTCAG-3′ and 5′-GGATCCCAGCCAAGAACATCCCCAAAAGG-3′ and subcloned into pLV-CMVenh-EGFP vector, thus creating pLV-CMVenh-EGFP-Pri-mmu-miR-31 which was confirmed by sequencing. The vector was microinjected as a transgene into fertilized C57BL/6J mouse oocytes, and the transgenic offspring were screened for EGFP by PCR using the primers 5′-ATCACCAGAACACTCAGTGG-3′ and 5′-ACTCGGCGTAGGTAATGTCC-3′ at Shanghai Biomodel Organism Science and Technology Development Co. Transgenic offspring were backcrossed to C57BL/6J background for more than seven generations, and the mice were characterized clinically and histologically with age-matched, non-transgenic littermates. Two out of five founders were selected for further breeding and experiments according to the expression levels of miR-31 in peripheral blood mononuclear cells. miR-31^TG^ mice did not develop any skin phenotypes at basal conditions on either strain of mice at least for 6 months.

### Histological analysis and immunohistochemistry

After treatment of mice with IMQ, the mouse back skin was fixed in formalin and embedded in paraffin. Sections (6 μm) were stained with haematoxylin and eosin (H&E). Epidermal hyperplasia (acanthosis) and the number of dermal infiltrating cells were assessed as histological features. Briefly, for measuring acanthosis, the epidermal area was outlined, and its pixel size was measured using the lasso tool in Adobe Photoshop CS4. The relative area of the epidermis was calculated using the formula as follows: area=pixels/(horizontal resolution × vertical resolution). For counting dermal infiltrating cells, nine areas (4/9 square inch for one) in three sections of each sample were randomly taken, in which the number of infiltrating cells were calculated. For immunohistochemistry, Ki67 expression was evaluated in skin sections using rabbit anti-mouse Ki67 Ab (1:100), following the manufacturer's instructions.

### IL-23-mediated mouse of psoriasis

IL-23-mediated mouse model of psoriasis was established as previously published^45^. Generally, mouse ears were injected i.d. with 1 μg rmIL-23 (R&D Systems, #1887-ML-010) dissolved in 25 μl PBS into one ear and 25 μl PBS into the contralateral ear. Injections were continued every other day for 8 days (days 1, 3, 5 and 7). Ears were used for histological analysis on day 8.

### RNA-binding protein immunoprecipitation

Immunoprecipitation of RNA-binding protein–RNA complexes was performed using Magna RIP RNA-binding Protein Immunoprecipitation Kit (Millipore, #92590) according to manufacturer's instructions. In brief, epidermal cells were isolated after mice were treated with IMQ or vehicle cream. Keratinocytes were then lysed by RIP lysis buffer and stored at −80 °C. Magnetic beads were incubated with Ab (5 μg) with rotation for 30 min at RT. Anti-Ago2 (Abcam, #ab32381) was used as the Ab of interest, while anti-SNRNP70 and negative control normal rabbit IgG served as controls. After thrawn quickly and centrifuged at 14,000 r.p.m. for 10 min, the supernatant was incubated with beads–Ab complex for overnight at 4 °C. Immunoprecipitated RNA was analysed by qPCR. The primer sequence of ppp6c and miR-31 were 5′-CCGCTGGATCTGGACAAGTAT-3′, 5′-ACACTGGCTGAACATTCGACT-3′; 5′-GCAGTGGAAAGGTTCAGTGC-3′, 5′-TATCCTCAACCCTCCGTGTC-3′.

### Cell cycle analysis of keratinocytes

Primary murine keratinocytes were transfected with siRNA-410 and siRNA-NC (20 μM) by TurboFect or NHEK were treated with different recombinant cytokines (all from BD) as indicated. Cells were harvested after 24 or 48 h and cold 70% ethanol was gently added drop-wise for fixation, followed by resuspension in a blocking solution (2% BSA, 5% FBS, 0.2% Triton X-100 and 0.1% sodium azide) and incubated at 4 °C for 10 min. Cells were then pelleted and resuspended in propidium iodide solution (0.1 mg ml^−1^, Calbiochem/EMD). Flow cytometry was used to analyse cell cycle.

### *In vivo* administration of Ppp6c shRNA lentivirus

DNA oligonucleotides containing mouse ppp6c shRNA sequences designed with the TRC shRNA Design online tool from the RNAi Consortium (http://www.broadinstitute.org/rnai/public/resources/rules) were synthesized then subcloned into pLVX-shRNA2 vector (Clontech, #632179). Tested in NIH3T3 cell line, the shRNA sequences with the highest knockdown efficiency were as follows: 5′-GATCCGCCAAAGTTATTCCGAGCAGTTTTCAAGAGAAACTGCTCGGAATAACTTTGGTTTTTTACGCGTG-3′; 5′-AATTCACGCGTAAAAAACCAAAGTTATTCCGAGCAGTTTCTCTTGAAAACTGCTCGGAATAACTTTGGCG-3′. For production of lentivirus, 293FT cells were seeded at a density of 4 × 10^6^ cells per 10-cm dish 1 day before transfection. Cells were transfected by calcium phosphate treatment with 5 μg pMD2.G, 10 μg psPAX2 and 15 μg pLVX-shppp6c or pLVX-shRNA2 empty vector. Sixteen hours post transfection, cells were treated with 10 mM sodium butyrate for 12 h. Another 24-h cell incubation in complete medium was performed before the viral supernatant was harvested, passed through 0.45 μm filters to remove cellular debris and ultracentrifuged to concentrate the virus. Viruses were titered by infecting 293FT cells with serial dilutions in medium supplemented with 5 μg ml^−1^ Polybrene, and the green fluorescent protein-positive cells were counted by flow cytometry 48 h post infection. The transduction titres were between 1 × 10^8^ and 2 × 10^8^ TU ml^−1^ for concentrated virus preparations. A single dose of 100 μl shRNA-ppp6c or control lentivirus preparation was injected i.d. into the shaved dorsal skin of 8-week-old C57BL/6J female mice. These mice were treated with IMQ for 7 days before being killed to assess the severity of psoriasiform lesions histologically.

### BrdU incorporation assay

BrdU (Sigma, #B5002) was directly added to the culture medium to achieve a final concentration of 10 μM and cells were incubated for 1 h in CO_2_ incubator at 37 °C. Cells were then harvested and fixed with 5 ml pre-chilled 70% ethonal drop by drop while vortexing, followed by incubation at room temperature for 20 min. Afterwards, cells were washed and resuspended in 2 ml 2 N HCl, then incubated at RT for another 20 min. About 2 ml of 0.1 M Na_2_B_4_O_7_ was used to neutralize the residual HCl for 2 min after cells were washed with PBS. Finally, cells were stained with APC-anti-BrdU Ab (BioLegend, #339808), resuspended in propidium iodide (Life Technologies, #S10274) buffer and analysed with a BD FACSCanto II Flow Cytometer.

### *In vitro* keratinocyte wound assay

NHEK were grown to >90% confluence on RTC-coated six-well CellBIND tissue culture plates in medium. Artificial wounds were created by scraping the monolayers with a sterile 10 μl tip, and the cells were washed with PBS three times to remove floating cells. The NHEK monolayers were treated with or without IL-6 (50 ng ml^−1^). The healing rate was quantified using computer-assisted measurements of sequential phase contrast digital photomicrographs of the same 10 non-overlapping regions per well.

### Three-dimensional cell culture

Human dermal fibroblasts (5 × 10^5^ per well, Sciencell, #2320) were mixed with a neutralized type I collagen gel (Shengyou Biotechnology, Hangzhou, China, #200110-50) in accordance with the manufacturer's instructions. The mixed solution was aliquoted into a six-well plate (3 ml per well) and allowed to harden for 30 min in a CO_2_ incubator at 37 °C. HaCaT cells were then dispensed onto each gel at a density of 3 × 10^6^ in 3 ml of complete three-dimensional (3D) culture medium and incubated overnight. The complete 3D culture medium consisted of a 1:1 mixture of DermaLifeK complete medium (Lifeline Cell Technology, #LL-0007) and Fibroblast Medium (Sciencell, #2301), supplemented with 10% FBS and 1% penicillin–streptomycin solution. The following day, the hardened gels were detached from the plates and incubation was continued for 1 week until the size of the contracted gel stabilized. Cell strainers (Falcon, #352350) were placed upside down into a new six-well plate after removal of the strainer handles, and the 3D culture medium with or without IL-6 at a concentration of 50 ng ml^−1^ was poured into the wells until the nylon mesh of the strainers was covered. The contracted gel discs were then placed on the mesh so that the HaCaT cells lay on top of the gel discs and the fluid level was adjusted to just below the upper edge of the gel. It was important to ensure the gel was steeped in sufficient amount of fluid with its surface exposed to air. Half of the culture fluid was replaced with fresh medium every other day throughout the experiment.

### Measurement of epidermal thickness after 3D culture

Sections of 3D cultured cells stained with H&E were examined. The thickness of the epidermal layer was measured using Axio Cam HRc and Adobe Photoshop CS4. Six points were selected at random and measured from the basal layer to the horny layer on vertical sections.

### *In vivo* miR-31 interference

AntagomiR-31 with sequences complementary to mature miR-31, complete 2′-O-methylation of sugar, phosphorothioate backbone and a cholesterol-moiety at 3′-end were synthesized by RIBOBIO (Guangzhou, China). Antagomir-31 or NC (10 nmol) was injected i.d. for 3 consecutive days. Mice were then treated with IMQ. After treatment, skin samples were removed and preserved in liquid nitrogen.

### Statistical analysis

The data were analysed with GraphPad Prism 5 and are presented as the mean±s.e.m. Student's *t*-test was used when two conditions were compared, and analysis of variance (ANOVA) with Bonferroni or Newman–Keuls correction was used for multiple comparisons. Simple linear regression model was used to analyse the correlation between RNA levels of IL-6 and miR-31. Probability values of <0.05 were considered significant; two-sided Student's *t*-tests or ANOVA were performed. *P<0.05; **P<0.01; ***P<0.001; NS, not significant. Error bars depict s.e.m.

## Additional information

**How to cite this article:** Yan, S. *et al.* NF-κB-induced microRNA-31 promotes epidermal hyperplasia by repressing protein phosphatase 6 in psoriasis. *Nat. Commun.* 6:7652 doi: 10.1038/ncomms8652 (2015).

## Supplementary Material

Supplementary Information Supplementary Figures 1-13 and Supplementary Table 1
