# Supplementary material for: NF-κB-induced microRNA-31 promotes epidermal hyperplasia by repressing protein phosphatase 6 in psoriasis
Source: Nat Commun. 2015 Jul 3;6:7652. doi: 10.1038/ncomms8652 (PMC4506511; doi:10.1038/ncomms8652)
Supplement: Supplementary Information — Supplementary Figures 1-13 and Supplementary Table 1 [file ncomms8652-s1.pdf]

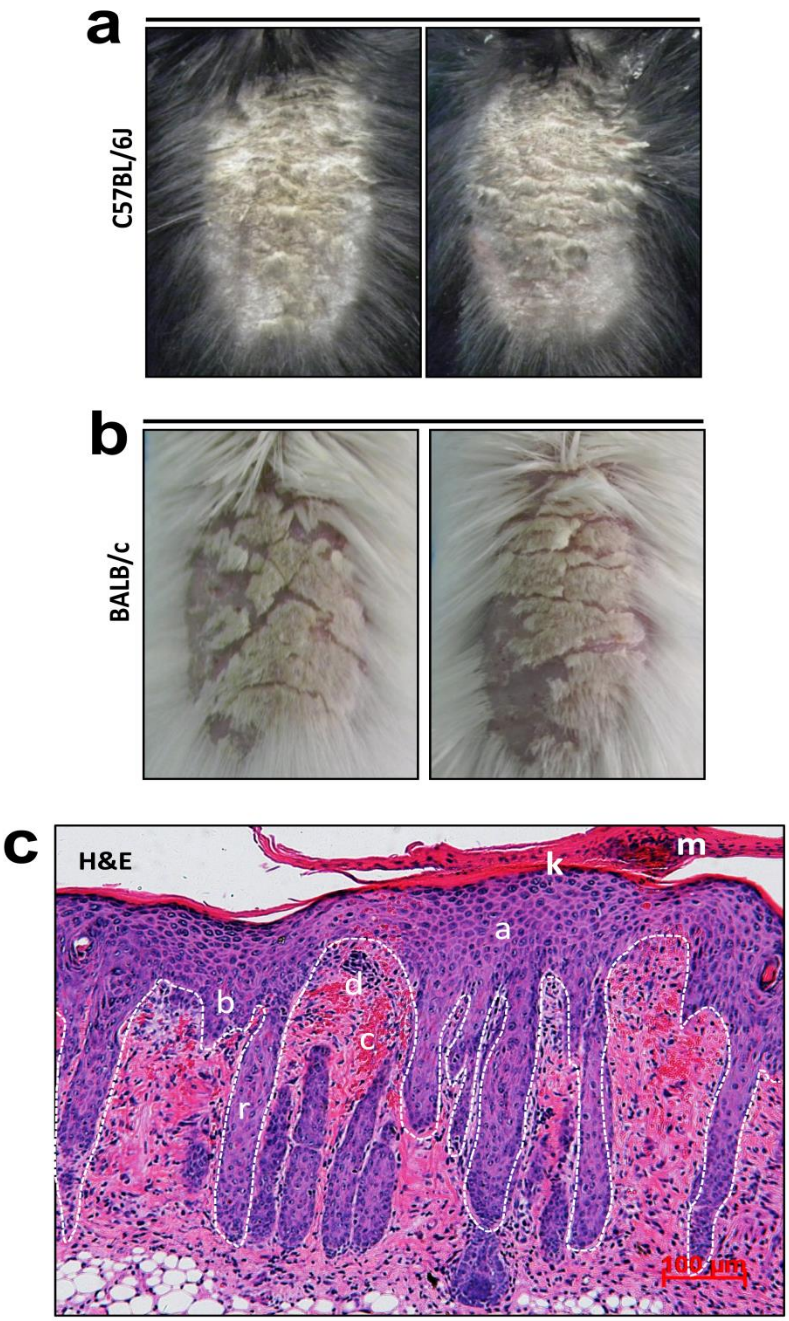

Supplementary Figure 1 IMQ-Induced Mouse Model of Psoriasis. IMQ cream was painted on the shaved back skin of C57BL/6J and BALB/c mice for 7 consecutive days. (**a, b**) Phenotypic presentation of mouse back skin treated with IMQ (severe erythema, scales and crusts). (**c**) H&E staining of lesional skin from treated mice. a, acanthosis; b,

increased proliferative basal layer epidermal keratinocytes; c, dilated capillaries (small blood vessels in dermis); d, dermal cell infiltrates; k, hyperkeratosis; m, microabscesses; r, elongated rete ridge. Dotted line indicates the border between the epidermis and dermis; scale bar, 100μm.

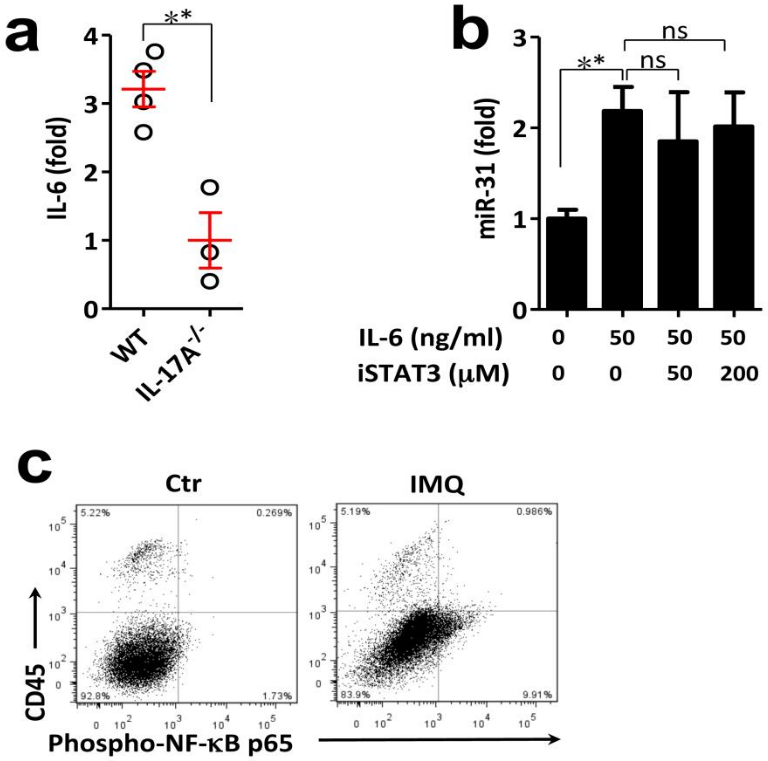

Supplementary Figure 2 Decreased IL-6 levels in IL-17A$^{-/-}$ mice and IL-6-mediated activation of NF-κB Signaling in Keratinocytes. (**a**) qPCR analysis of IL-6 expression from skin of WT (n=4) and IL-17A$^{-/-}$ mice (n=3) treated with IMQ. Results are presented as the ratio of mRNA to β-actin, relative to that in IL-17A$^{-/-}$ mice. (**b**) miR-31 expression in HaCaT cells stimulated with IL-6 in absence or presence of various concentrations of STAT3 inhibitor (iSTAT3) (S3I-201, Selleck #S1155). Results are presented as the ratio of miRNA to the small nuclear RNA U6, relative to that in untreated cells. (**c**) Phosphorylated-p65 (P-p65) and CD45 flow cytometry analysis of single cell suspensions from normal epidermis of mouse treated with vehicle (Ctr) or lesional epidermis of mouse treated with IMQ. **$p<0.01$, two-tailed Student's *t*-test. Error bars depict SEM.

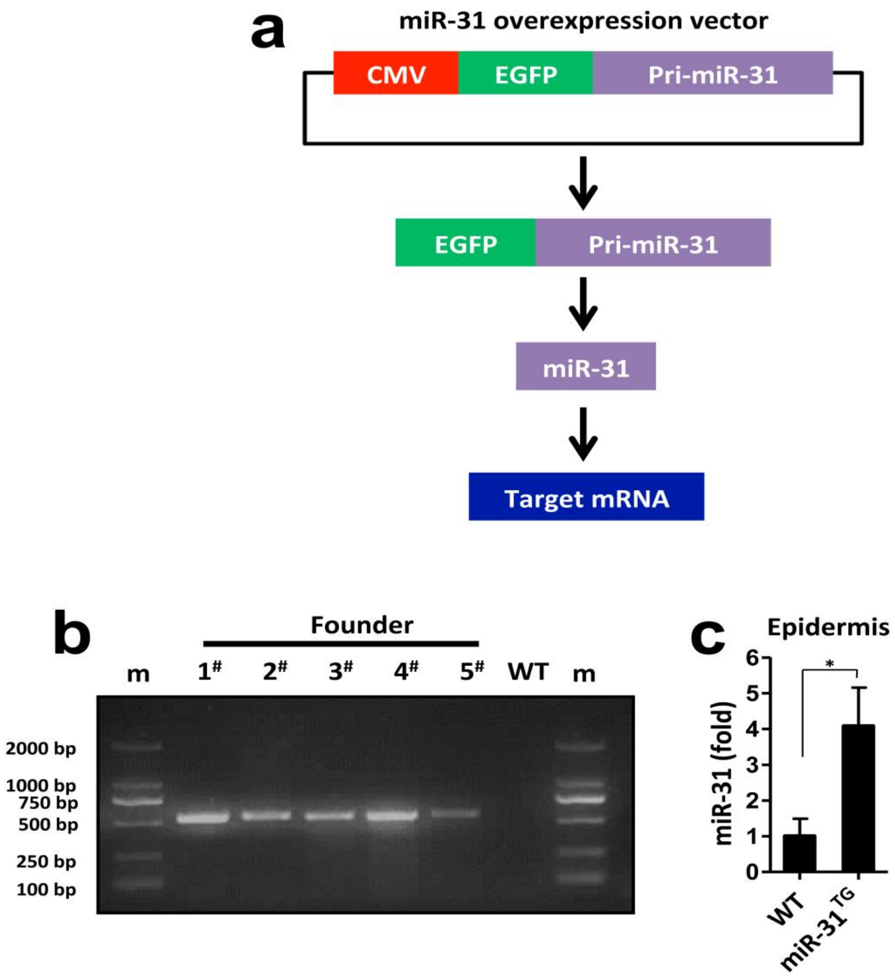

Supplementary Figure 3 Generation of miR-31^{TG} Mice. (**a**) Schematic illustration of the miR-31 transgenic construct used in this study to overexpress miR-31. CMV, cytomegalovirus (promoter); EGFP, enhanced green fluorescent protein. (**b**) PCR genotyping of miR-31 transgenic founder mice (533bp). $^{\#}$indicated genotyping positive founder lines; m, DNA size marker; WT, wild-type control. (**c**) Quantitative RT-PCR analysis of miR-31 in PBMCs derived from WT or miR-31^{TG} mice (n=7 for each group). Results are presented as the ratio of miRNA to the small nuclear RNA U6, relative to that in WT mice. *$p<0.05$, two-tailed Student's *t*-test. Error bars depict SEM.

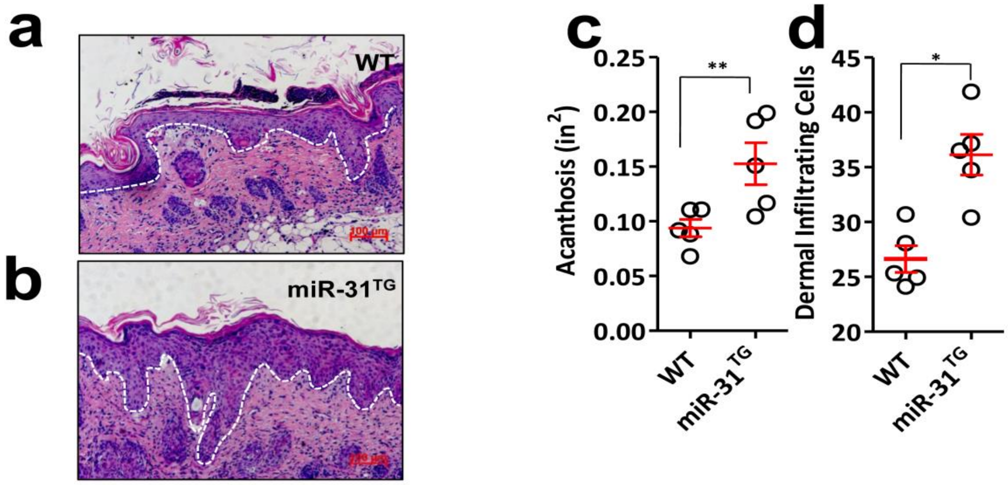

Supplementary Figure 4 Increased Disease Severity in miR-31^{TG} Mice Treated with IMQ. (**a, b**) H&E staining of the back skin of WT or miR-31^{TG} mice treated with IMQ. (**c**) Digital photos of H&E-stained skin samples derived from WT or miR-31^{TG} mice treated with IMQ were taken at the same orientation and magnification (n=5). The epidermal area was outlined, and its pixel area was measured using the lasso tool in Adobe Photoshop CS4. The relative area of the epidermis was calculated by the formula provided in Methods. Scale bar, 100μm. (**d**) Dermal cell infiltrates for WT or miR-31^{TG} mice treated with IMQ. For all measurements, the median number of specifically stained dermal nucleated cells was counted in 3 high-power fields per section. *p<0.05, **p<0.01, two-tailed Student's *t*-test. Error bars depict SEM.

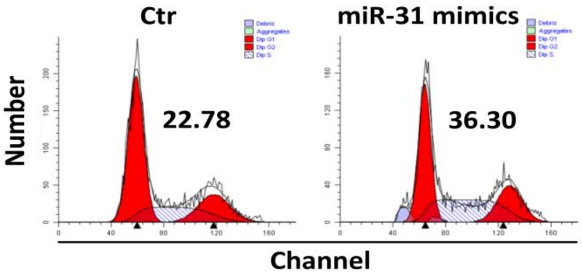

Supplementary Figure 5 Enhanced Proliferation of NHEK after overexpressing miR-31. Cell cycle analysis of NHEK transfected with control mimics (Ctr) and miR-31 mimics. Data are representative of two independent experiments.

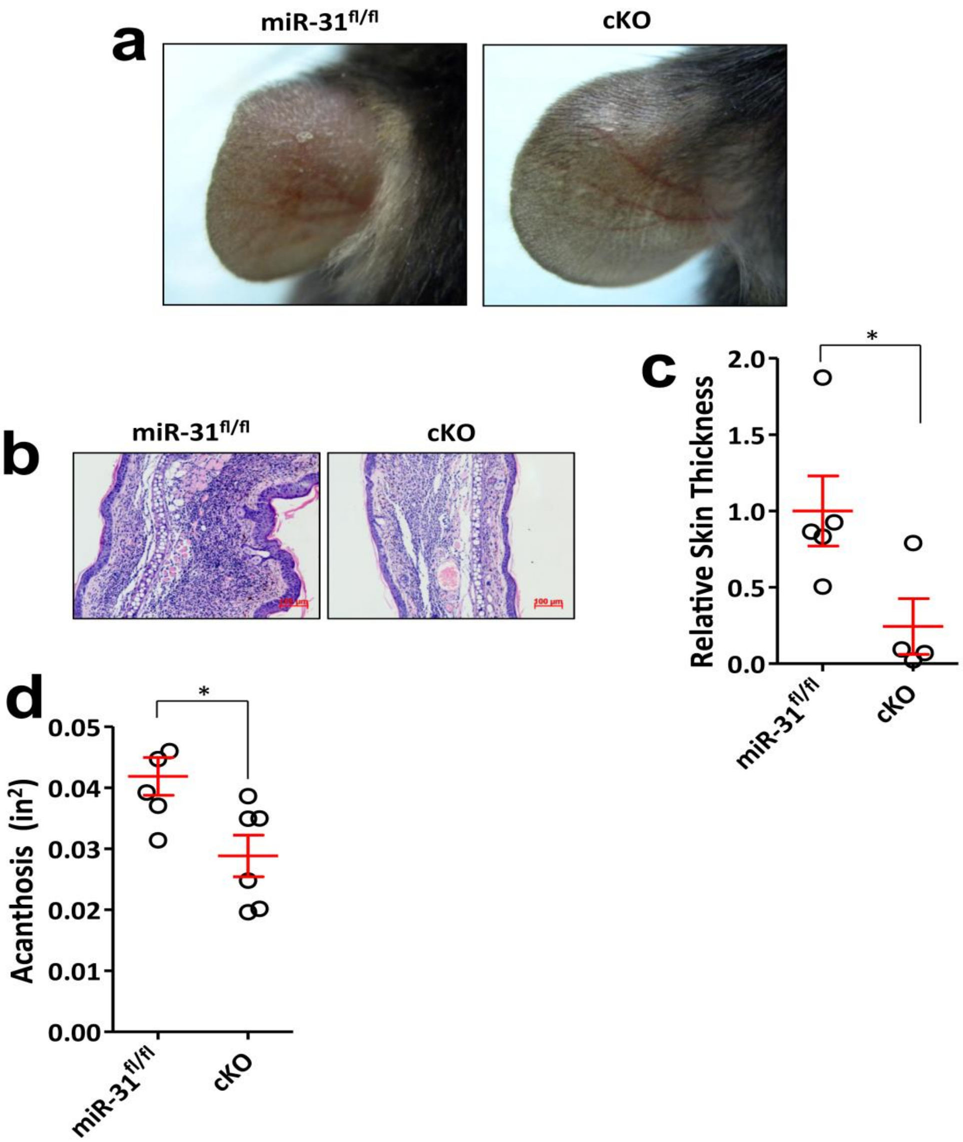

Supplementary Figure 6 Decreased Disease Severity in cKO mice after IL-23 Treatment. Mouse recombinant IL-23 was injected intradermally into the ear skin of miR-$31^{fl/fl}$ (n=5) and cKO mice (n=4) every other day for 14 days. (**a**) Phenotype of ear skin from miR-$31^{fl/fl}$ and cKO mice treated with rIL-23. (**b**) H&E staining of lesional skin from miR-$31^{fl/fl}$ and cKO mice treated with rIL-23. Scale bar, 100μm. (**c, d**) Skin thickness and acanthosis were quantitated from the H&E staining. *p<0.05, two-tailed Student's *t*-test. Error bars depict SEM.

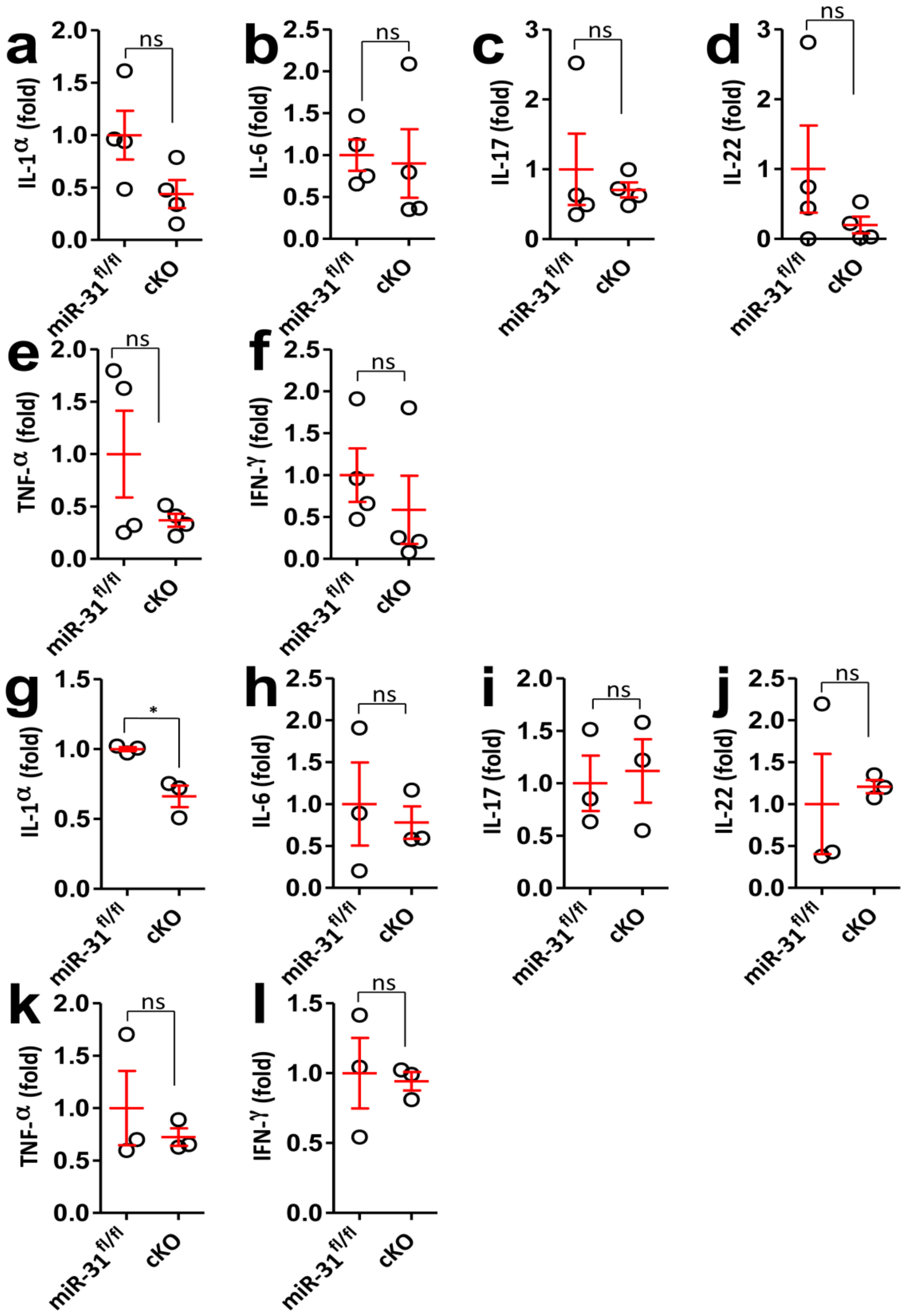

Supplementary Figure 7 Expression Levels of Inflammatory Genes in Lesional Skin of IMQ-Induced and IL-23-Mediated Mouse Models of Psoriasis. (**a-f**) Expression levels of IL-1α, IL-6, IL-17A, IL-22, IFN-γ and TNF-α in lesional skin samples derived from either miR-31$^{fl/fl}$ or cKO mice treated with IMQ. (**g-l**) Expression levels of IL-1α,

IL-6, IL-17, IL-22, IFN-γ and TNF-α in lesional ear skin samples derived from either miR-31$^{fl/fl}$ or cKO mice treated with rIL-23. Data are representative of two independent experiments. ns, not significant, *p<0.05, two-tailed Student's *t*-test. Error bars depict SEM.

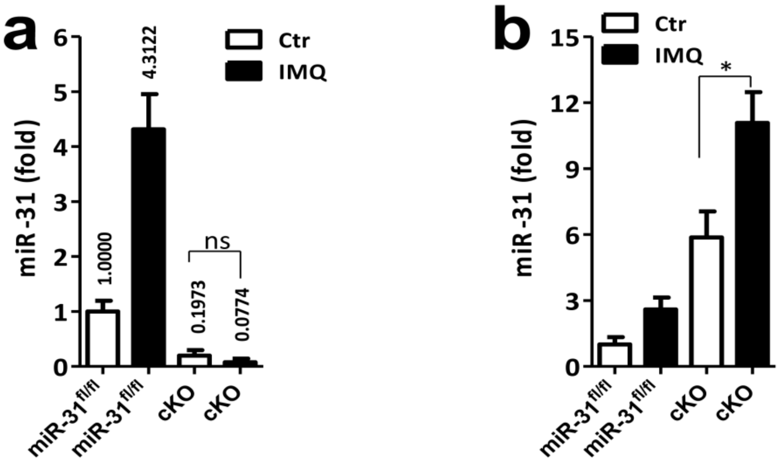

Supplementary Figure 8 miR-31 Expression in Epidermal Cells and Splenocytes of miR-31$^{fl/fl}$ and cKO Mice Treated with IMQ. (**a**) Expression of miR-31 in epidermis from miR-31$^{fl/fl}$ and cKO mice treated with vehicle (Ctr) or IMQ (n=5 for each group). (**b**) Expression of miR-31 in splenocytes from miR-31$^{fl/fl}$ and cKO mice treated with vehicle (Ctr) or IMQ (n=5 for each group). Results are presented as the ratio of miRNA to the small nuclear RNA U6, relative to that in miR-31$^{fl/fl}$ mice treated with vehicle. ns, not significant, *$p<0.05$, two-tailed Student's *t*-test. Error bars depict SEM.

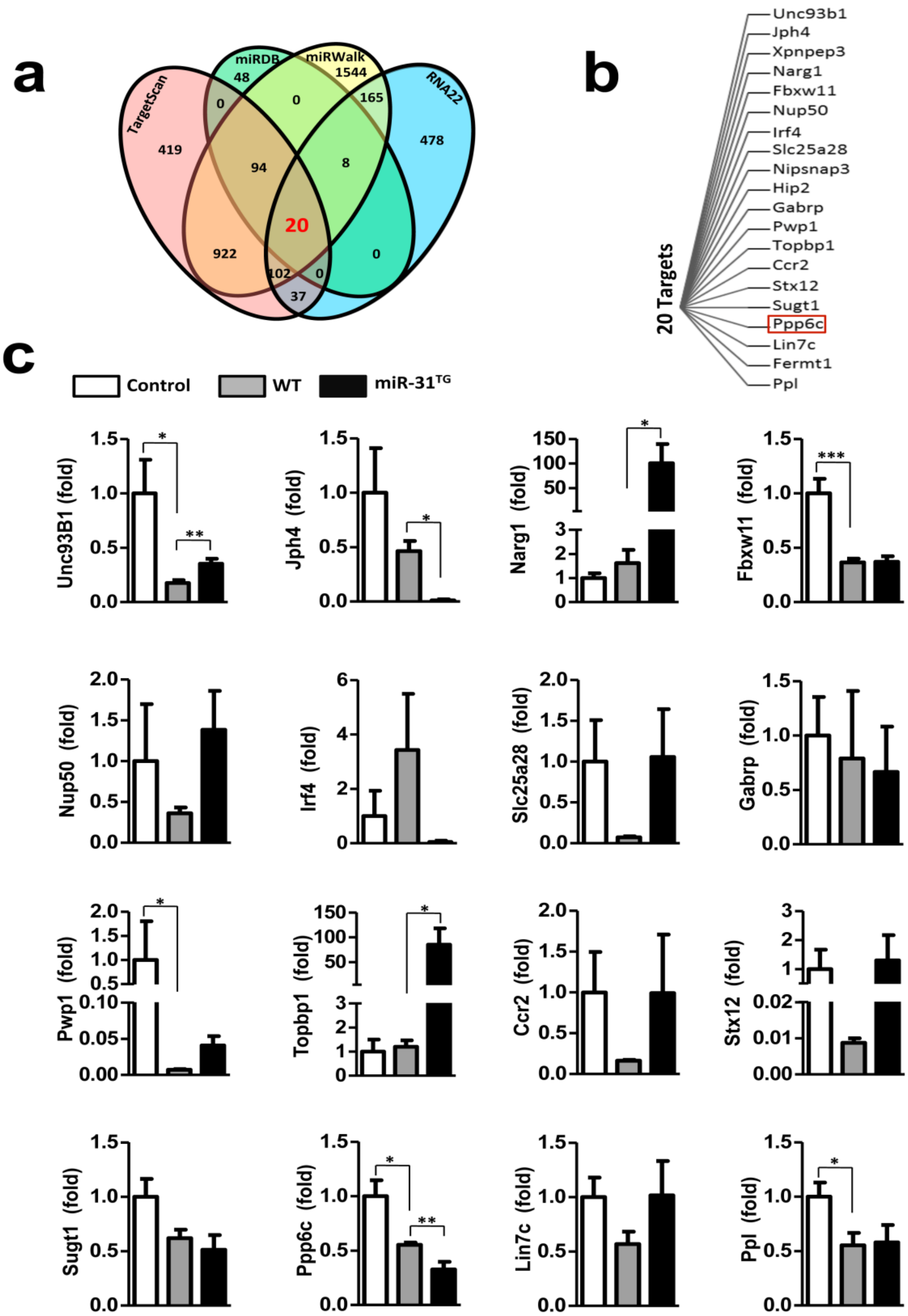

Supplementary Figure 9 Identification of miR-31 Target Gene(s). (**a, b**) Predicted 20 candidate targets of miR-31. RNA was extracted from skin samples of non-treated WT mice (Control), WT mice treated with IMQ (WT) and miR-31^{TG} mice treated with IMQ (miR-31^{TG}). (**c**) mRNA levels of 16 candidate genes were measured by qPCR, and the

rest 4 genes were undetectable. Results are presented as the ratio of mRNA to β-actin, relative to that in untreated WT controls. *$p<0.05$, **$p<0.01$, ***$p<0.001$, two-tailed Student's *t*-test. Error bars depict SEM.

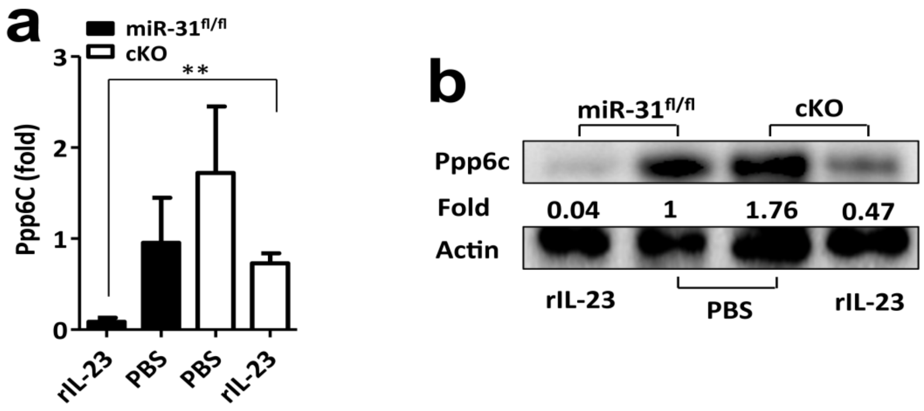

Supplementary Figure 10 Upregulated Ppp6c Levels in cKO Mice in IL-23-mediated Mouse Model of Psoriasis. (**a**) qPCR analysis of ppp6c expression in ear skin of miR-31$^{fl/fl}$ and cKO mice, treated with rIL-23 or PBS (n=4-5). Results are presented as the ratio of mRNA to β-actin, relative to that in miR-31$^{fl/fl}$ mice treated with PBS. (**b**) Western blotting of ppp6c expression in ear skin of miR-31$^{fl/fl}$ and cKO mice, treated with rIL-23 or PBS. Data are representative of two independent experiments. **p<0.01, two-tailed Student's *t*-test. Error bars depict SEM.

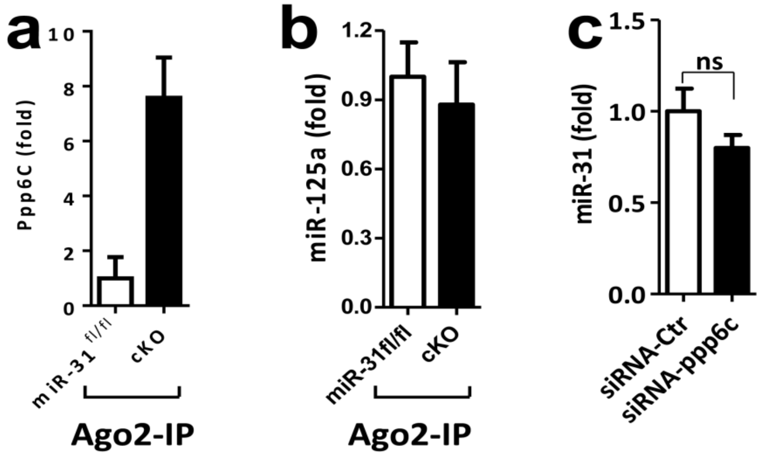

Supplementary Figure 11 Ppp6c is not Targeted by miR-125a and Silencing Ppp6c does not alter miR-31 expression. (**a, b**) Ago2 was immunoprecipitated from epidermis lysates derived from miR-31$^{fl/fl}$ or cKO mice treated with IMQ. Immunoprecipitates were assayed for ppp6c and miR-125a. (**c**) Expressin of miR-31 in NHEK transfected with non-targeted siRNA (siRNA-Ctr) and ppp6c targeted siRNA (siRNA-ppp6c). Results are presented as the ratio of mRNA to β-actin, relative to that in NHEK transfected with non-targeted siRNA. Data are representative of two independent experiments. ns, not significant, two-tailed Student's *t*-test. Error bars depict SEM.

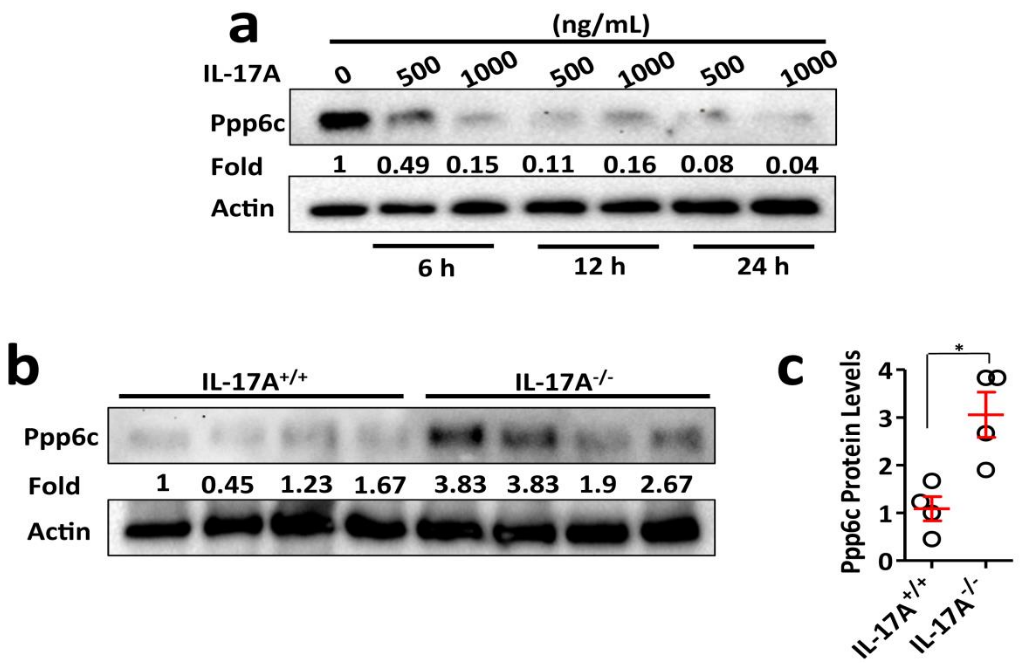

Supplementary Figure 12 IL-17A Inhibits Ppp6c Expression in Keratinocytes. (**a**) Western blotting of ppp6c expression in primary mouse keratinocytes stimulated by IL-17A with different dosages and time points. (**b, c**) Western blotting of ppp6c expression in epidermis derived from IL-17A$^{+/+}$ or IL-17A$^{-/-}$ mice treated with IMQ for 7 days. Values (**a, b**) were expressed as fold changes relative to non-stimulated keratinocytes (**a**) or to IL-17A$^{+/+}$ mouse (**b**). **p*<0.05, two-tailed Student's *t*-test. Error bars depict SEM.

Fig. 2h

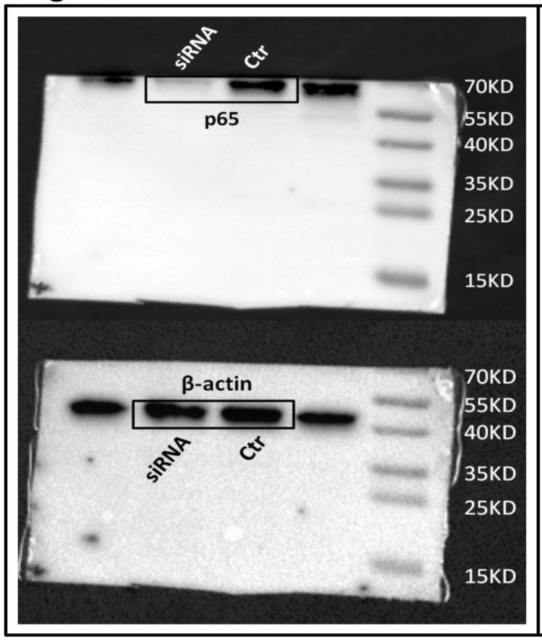

Fig. 4b

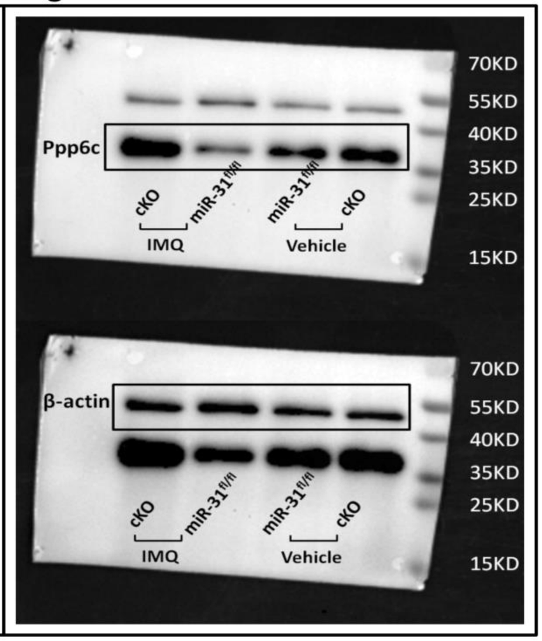

Fig. 5a

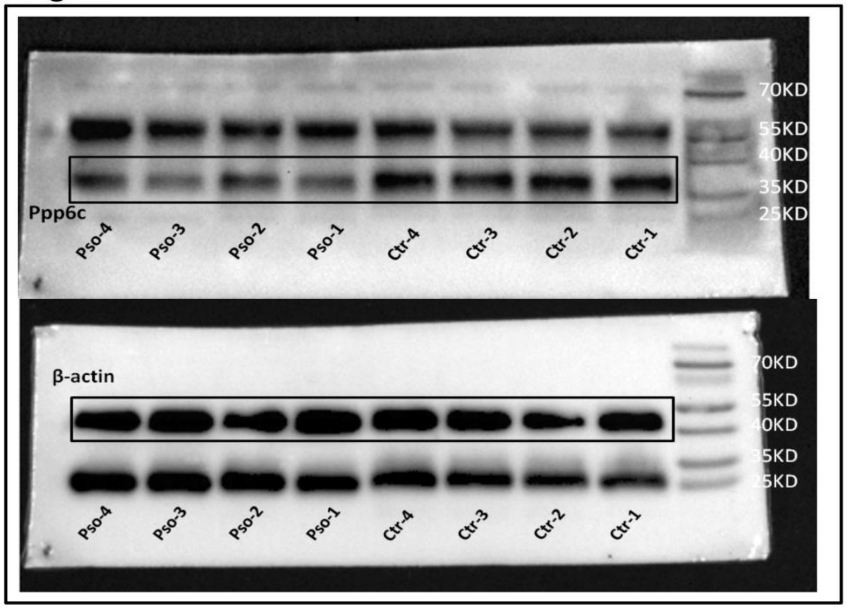

Fig. 5d

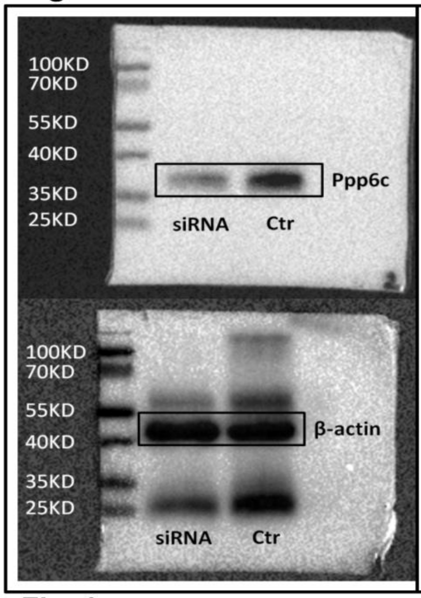

Fig. 5f

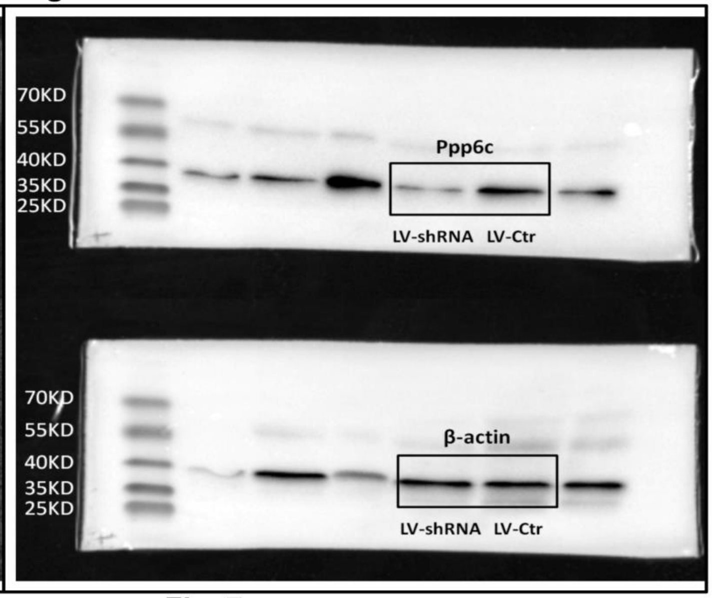

Fig. 6g

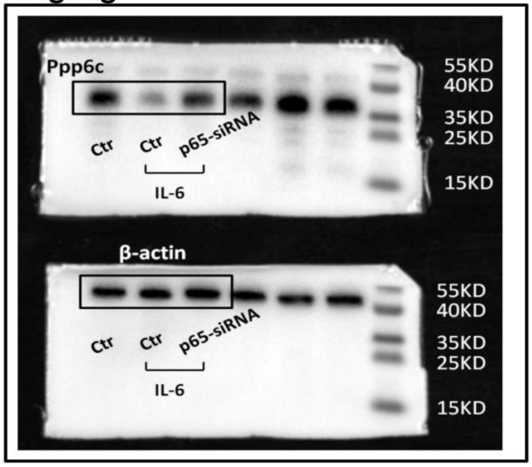

Fig. 7e

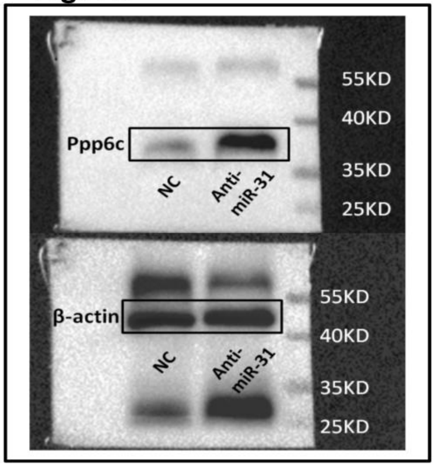

Sup Fig. 10b

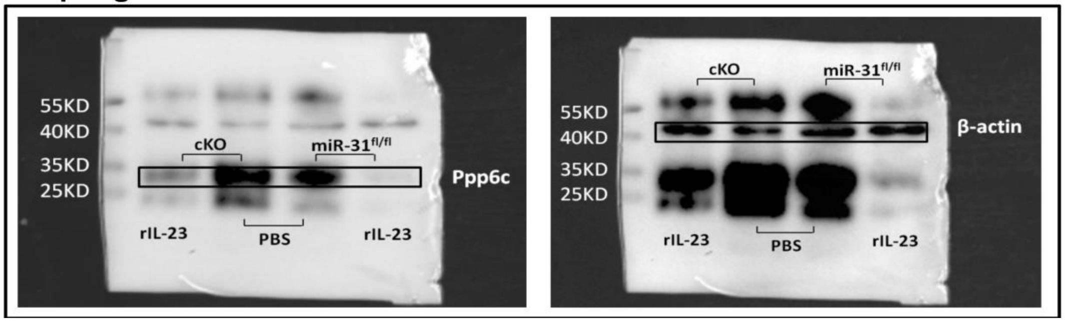

Sup Fig. 12a

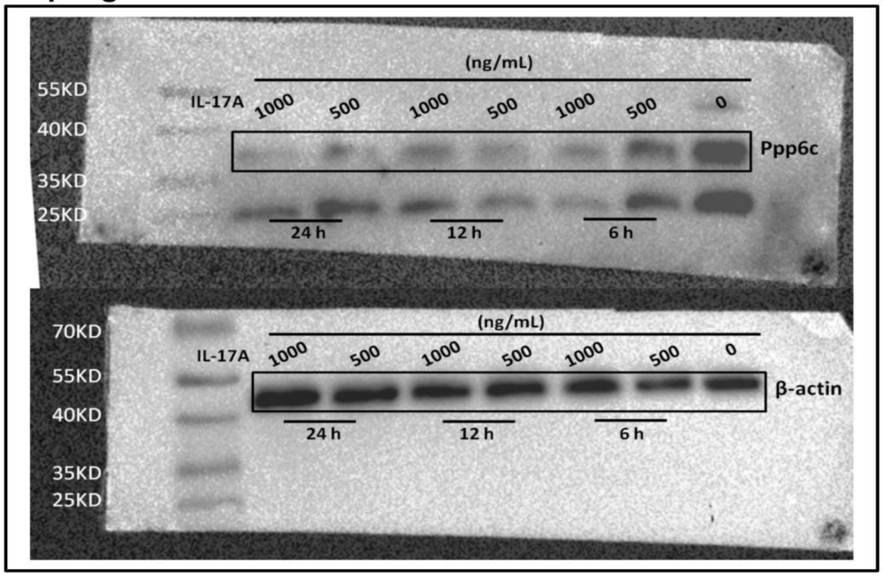

Sup Fig. 12b

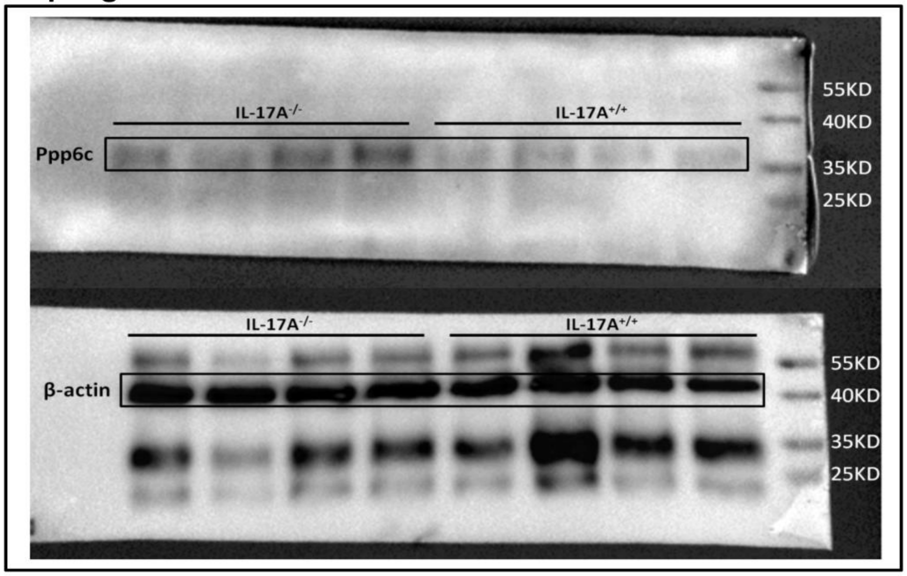

Supplementary Figure 13 List of original pictures of western blots. Black boxes highlight the indicated lanes in figures.

Supplementary Table 1. Information for patients with psoriasis vulgaris

Sample ID	Age in Years	Gender	PASI Score
**1**	31	M	22.2
**2**	57	M	16.7
**3**	62	M	18.2
**4**	28	M	10.4
**5**	55	M	15
**6**	19	F	16.8
**7**	35	F	21.4
**8**	41	M	unknown
**9**	33	M	13.6
**10**	24	F	7.8
**11**	38	M	9.2
**12**	52	M	unknown
**13**	43	M	17.1
**14**	28	M	16.0
**15**	36	M	unknown
**16**	34	F	13.9
**17**	72	M	16
**18**	45	M	7.2
**19**	47	F	10.6
**20**	37	M	15.2
**21**	28	M	10.4
**22**	35	M	12.8
**23**	74	F	11.6
**24**	54	M	7.4
**25**	39	M	14.5
**26**	67	M	3.9
**27**	63	M	16.8
**28**	59	M	3.2
**29**	87	M	14.9

All patients were clinically diagnosed as psoriasis vulgaris. M, male; F, female; PASI, Psoriasis Area and Severity Index; Unknown, data was missing.
